# Let Us Talk Money: Subjectively Reported Financial Performance of People Living with Neurodegenerative Diseases—A Systematic Review

**DOI:** 10.1007/s11065-023-09597-0

**Published:** 2023-08-18

**Authors:** Akke-Marij D. Ariesen, Roosa E. Tuomainen, Peter P. De Deyn, Oliver Tucha, Janneke Koerts

**Affiliations:** 1 Department of Clinical and Developmental Neuropsychology, University of Groningen, Groningen, The Netherlands; 2 Department of Neurology and Alzheimer Center Groningen, University Medical Center Groningen, Groningen, The Netherlands; 3 Laboratory of Neurochemistry and Behaviour, University of Antwerp, Antwerp, Belgium; 4 Department of Neurology and Memory Clinic, Middelheim General Hospital (ZNA), Antwerp, Belgium; 5 Department of Psychiatry and Psychotherapy, University Medical Center Rostock, Rostock, Germany; 6 Department of Psychology, Maynooth University, National University of Ireland, Maynooth, Ireland

**Keywords:** Financial performance, Neurodegenerative diseases, Mild cognitive impairment, Alzheimer’s disease, Parkinson’s disease, Multiple sclerosis

## Abstract

Neurodegenerative diseases (NDDs) form a heterogeneous, widespread group of disorders, generally characterized by progressive cognitive decline and neuropsychiatric disturbances. One of the abilities that seems particularly vulnerable to the impairments in neurodegenerative diseases is the capability to manage one’s personal finances. Indeed, people living with neurodegenerative diseases were shown to consistently present with more problems on performance-based financial tasks than healthy individuals. While objective, performance-based tasks provide insight into the financial competence of people living with neurodegenerative diseases in a controlled, standardized setting; relatively little can be said, based on these tasks, about their degree of success in dealing with the financial demands, issues, or questions of everyday life (i.e., financial performance). The aim of this systematic review is to provide an overview of the literature examining self and informant reports of financial performance in people living with neurodegenerative diseases. In total, 22 studies were included that compared the financial performance of people living with mild cognitive impairment (MCI), Alzheimer’s disease (AD), Parkinson’s disease, or multiple sclerosis to a (cognitively) normal control group. Overall, the results indicate that people living with neurodegenerative diseases are more vulnerable to impairments in financial performance than cognitively normal individuals and that the degree of reported problems seems to be related to the severity of cognitive decline. As the majority of studies however focused on MCI or AD and made use of limited assessment methods, future research should aim to develop and adopt more comprehensive assessments to study strengths and weaknesses in financial performance of people living with different neurodegenerative diseases.

## Introduction

Neurodegenerative diseases, such as Alzheimer’s disease (AD), Parkinson’s disease (PD), Huntington’s disease, and multiple sclerosis (MS), form a heterogeneous, widespread group of disorders that can generally be characterized by a progressive decline of cognition and neuropsychiatric disturbances (American Psychiatric Association, 2013; Hardiman & Doherty, 2016; Simon, 2017). These disease characteristics can lead to functional impairments in complex, higher-order as well as more basic activities of daily life (American Psychiatric Association, 2013). One of the abilities that seems to be particularly vulnerable to the impairments in neurodegenerative diseases is the capability to manage one’s personal finances (Griffith et al., 2003; Marson et al., 2000; Martin et al., 2013; Sudo & Laks, 2017).

Being able to adequately handle financial tasks, such as paying bills, budgeting, or taking out insurance, is crucial for successful independent living. Difficulties in handling finances can have adverse personal and legal consequences (Engel et al., 2016; Triebel et al., 2018) and may lead to financial insecurity, debts, poverty, or even financial abuse (Dong et al., 2011; Engel et al., 2016; Manthorpe et al., 2012; Marson et al., 2000; Okonkwo et al., 2008). Despite this demonstrable importance in everyday life, financial capability has received relatively limited scientific attention in the context of neurodegenerative diseases. Specifically, clinically oriented research into financial capability is lacking, and most existing studies focus on dementia or AD rather than on other neurodegenerative diseases (e.g., Marson et al., 2000; Martin et al., 2013). Early recognition and evaluation of difficulties in financial capability is especially significant in people living with neurodegenerative disease, however, as it may enable researchers and clinicians to identify and timely offer the required type and level of support. Moreover, recent studies suggest that problems with certain aspects of financial capability, including financial decision-making and susceptibility to financial scams and exploitation, may reflect the accumulation of neurodegenerative pathology in the earliest stages of the disease process (i.e., prior to the onset of noticeable cognitive impairments) and could signal subsequent cognitive decline (Fenton et al., 2022; Kapasi et al., 2021). In line with this, it has been suggested that for some neurodegenerative diseases, a decline in financial capability could mark the progression from the mild cognitive impairment (MCI) stage to the dementia stage (Gerstenecker et al., 2016; Martin et al., 2008, 2013).

**Table 1:** Characteristics of financial competence measures and financial performance measures

**Financial competence measures**	**Financial performance measures**
Are often objective (performance-based)	Are often subjective (self or informant report)
Assess a construct that includes financial knowledge and financial judgment	Assess a broad construct that includes financial competence
Reflect functioning in a controlled environment	Reflect real-world functioning
Provide with standardized scores that are easily comparable across participants or groups	Can take the contextual factors in one’s personal environment into account
Often reflect optimal performance at the moment of assessment	Often reflect typical performance over longer periods of time
Address an individual’s performance during a financial task	Require the individual or informant to recall how well they usually perform or have performed everyday financial tasks
Include objective scoring of financial skills, making them less susceptible to bias	Are susceptible to inaccuracy and reporter bias (i.e., over-reporting and under-reporting)
Are generally independent of the level of insight of the individual into current daily-life functioning	Require adequate insight of the individual or informant into everyday financial functioning
Often require a relatively large time investment of the examiner and examinee (i.e., relatively high participant burden)	Can be relatively easily applied in a clinical or daily-life setting (e.g., require less time, can be filled in by an informant)

**Table 2:** Overview of instruments, subscales, and items on financial performance included in this systematic review

**Instrument**	**Instrument description**	**Item(s)/subscale(s) for financial performance **	**Scoring/outcome measures of financial performance**	**Psychometric properties**	**Used in/modifications**
The Aging and Memory Quality of Life survey (AMQoL; Gibson et al., 2020)	- Self and informant report survey developed to detect adaptive and maladaptive behaviors that follow an MCI diagnosis - Target population: people living with MCI - Consists of 39 closed questions in four categories: 1. Biological/health care questions; 2. Psychological questions; 3. Social questions; **4. Legal/economic**	**“Do you want help with your finances?”** **“Have others helped you with your finances?”** - The items are coded as positive (adaptive) or negative (maladaptive) behaviors. Both finances-related items were coded as positive	- People living with MCI are asked to think about the items “since their MCI was diagnosed.” - CNCs are asked to think about the items regarding their experiences with aging - An affirmative response to the finance-related items reflects worse performance	- Developed by clinicians; validated through interviews with people living with MCI - Psychometric properties remain to be explored	Gibson et al. (2020)
The Alzheimer’s Questionnaire (AQ; Sabbagh et al., 2010)	- Informant report assessment used for the diagnosis of dementia - Target population: people living with cognitive impairment - Consists of 21 items in five domains: 1. Memory; 2. Orientation; **3. Functional ability;** 4. Visuospatial; 5. Language	**“Excluding physical limitations (e.g., tremor, hemiparesis, etc.), does the patient have trouble handling money (tips, calculating change?)”** **“Excluding physical limitations (e.g., tremor, hemiparesis etc.), does the patient have trouble paying bills or doing finances; OR are family members taking over finances because of concerns about ability?”**	- Items are presented in a yes/no format, where “yes” indicates worse performance - A lower (total) score reflects better performance - Along with five other AQ items, the second finance-related item is a good predictor of AD diagnosis and is therefore weighted more heavily into the total score	- Internal consistency: good (Cronbach’s *α* = 0.89) - Sensitivity: excellent (MCI = 89%; AD = 99%) - Specificity: excellent (MCI = 91%; AD = 96%) (Malek-Ahmadi et al., 2012a, b)	Malek-Ahmadi et al. (2012a)
The Current Financial Capacity Form (CFCF; Wadley et al., 2003)	- Self and informant report scale used to assess a person’s current level of financial capacity - Target population: community-dwelling older adults - Consists of 18 items in eight domains: **1. Basic monetary skills;** **2. Financial conceptual knowledge;** **3. Cash transactions;** **4. Checkbook management;** **5. Bank statement management;** **6. Financial judgment;** **7. Bill payment;** **8. Knowledge of assets/estate;** **Global financial capacity (total score)**	**All 18 items relate to financial performance** Example items: **“Naming coins/currency”** **“Define financial concepts”** **“Understand bank statement”**	- Items are scored on a 3-point scale: (1) “can do without help,” (2) “can do with help,” (3) “cannot do even with help.” - A lower (total) score reflects better performance	- Test–retest reliability for the informants of people living with AD: acceptable (*r* = 0.71) - Test–retest reliability for informants of CNCs: excellent (*r* = 0.99) (Wadley et al., 2003)	Griffith et al. (2003) Gerstenecker et al. (2019): - Refer to the instrument as the CFAR - Used response options on a 4- point Likert scale: (1) “can perform alone without difficulty”; (2) “can perform with some difficulty”; (3) “can perform with a lot of difficulty”; (4) “cannot perform, even with help.” - Included a ninth domain called “Investment decisions.”
The Disability Assessment for Dementia scale (DAD; Gélinas et al., 1999)	- Informant report questionnaire used to evaluate cognitive functioning - Target population: people living with cognitive impairment - Consists of 40 questions in 10 domains: ADL: 1. Dressing; 2. Hygiene; 3. Continence; 4. Eating IADL: 5. Meal preparation; 6. Telephoning; **7. Taking care of finance and correspondence;** 8. Going on an outing; 9. Taking medications and ability to stay safely at home; 10. Leisure and housework Total DAD score	**“Show an interest in his/her personal affairs such as his/her finances and written correspondence”** **“Organize his/her finance to pay his/her bills (checks, bankbook, bills)”** **“Adequately organize his/her correspondence with respect to stationery, address, stamps”**^**a**^ **“Handle adequately his/her money (make change)”**	- Used to evaluate behavior from the past two weeks - Response options: (1) “yes,” (0) “no,” or “n/a.” - A higher (total) score reflects better performance	- Internal consistency: excellent (Cronbach’s *α* = 0.96) - Test–retest reliability: excellent (ICC = 0.96) - Interrater reliability: excellent (ICC = 0.95) (Gélinas & Gauthier, 1994)	Charernboon and Lerthattasilp (2016)
The Disability Assessment for Dementia Chinese version (C-DAD; Mok et al., 2005)	- Informant report questionnaire based on the original DAD by Gélinas et al. (1999) Target population: people living with dementia in the Chinese population - Consists of 47 questions in 11 subscales 1. Hygiene; 2. Dressing; 3. Continence; 4. Eating; 5. Meal preparation; 6. Telephoning; 7. Going on an outing; **8. Finance;** 9. Medications; 10. Housework; 11. Leisure	**“Show an interest in his/her personal affairs such as his/her finances”** **“Organize his/her finances to pay his/her bills”** **“Handle adequately his/her money (know the exact change)”**	- Used to evaluate behavior from the past two weeks - Response options: (1) “yes,” (0) “no,” or “n/a.” - A higher (total) score reflects better performance	- Internal consistency: excellent (Cronbach’s *α* = 0.91) - Internal consistency for the “Finance” subscale: questionable (Cronbach’s *α* = 0.63) - Test–retest reliability: excellent (ICC = 0.99) - Test–retest reliability for the “Finance” subscale: almost perfect (*κ* = 0.91) - Interrater reliability: excellent (ICC = 0.98) - Has been validated on cognitively normal Chinese elderly and people living with AD (Mok et al., 2005; Yeh et al., 2011)	Yeh et al. (2011)
The Functional Activities Questionnaire (FAQ; Pfeffer et al., 1982)	- Informant reported IADL measure to assess functional capacity of individuals - Target population: older adults - Consists of 10 forced-choice items	**“Writing checks, paying bills, balancing checkbook”** **“Assembling tax records, business affairs, or papers”**	- Items are rated from 0 (normal) to 3 (dependent) - A lower (total) score reflects better performance	- Internal consistency: excellent (Cronbach’s *α* = 0.98) - Interrater reliability: excellent (*r* = 0.97) - Test–retest reliability for total sample: excellent (ICC = 0.95), CNCs: moderate (ICC = 0.67) - Sensitivity: good (85%) - Specificity: good (81%) (González et al., 2021; Pfeffer et al., 1982)	Brown et al. (2011)
The Instrumental Activities of Daily Living – Compensation scale (IADL-C; Schmitter-Edgecombe et al., 2014)	- Informant report scale used to assess early functional difficulties and detect compensation strategies - Target population: older adults - Consists of 27 items in 10 categories, in four factors: **Factor 1. Money and self-management skills;** **1. Financial management;** 2. Household activities; 3. Shopping; 4. Organization; 5. Medication management Factor 2. Home daily living skills; 6. Meal preparation; 7. Phone use Factor 3. Travel and event memory skills; 8. Travel/driving Factor 4. Social skills; 9. Conversation; 10. Social activities	**“Keep financial records organized”** **“Can manage a budget and business affairs”** **“Balances a check book or credit card statement”** **“Remembers whether bills were paid”**	- Items are scored on an 8-point Likert scale from 1 (independent; as well as ever) to 8 (not able to complete activity anymore) - Informants can also respond either “does not need to complete the activity” or “no basis for judgment.” - A lower (total) score reflects better performance	- Internal consistency: good to excellent (Cronbach’s *α* = 0.80 to 0.93) - Test–retest-reliability: acceptable to excellent (*r* = 0.70 to 0.91) (Schmitter-Edgecombe et al., 2014)	Schmitter-Edgecombe et al. (2014)
The Korean Instrumental Activities of Daily Living (K-IADL; Won et al., 2002)	- Self-report measure developed to assess (I)ADL in the Korean population - Target population: older adults in Korea - Consists of seven ADL, and ten IADL items	**“Handling money”**	- Seven items (including handling money) are scored on a 4-point Likert scale - Other items are scored on a 3-point scale - A lower (total) score reflects better performance	- Internal consistency: excellent (Cronbach’s *α* = 0.96) - Test–retest reliability: excellent (*r* = 0.94) - Validity: acceptable: correlated significantly with measures of global cognition (e.g., MMSE, *r* = -0.73) - Sensitivity: good (83%) - Specificity: good (82%) (Kang et al., 2002)	Shin et al. (2015)
The Lawton – Brody Instrumental Activities of Daily Living scale (Lawton – Brody IADL; Lawton & Brody, 1969)	- Self and informant report instrument used to assess more complex independent living skills - Target population: older adults - Consists of eight domains: 1. Ability to use telephone; 2. Shopping; 3. Food preparation; 4. Housekeeping; 5. Laundry; 6. Mode of transportation; 7. Responsibility for own medications; **8. Ability to handle finances** - Can be used to assess decline/improvement over time	**“Ability to handle finances”** Response options: 1. Manages financial matters independently (budgets, writes checks, pays rent, bills, goes to bank), collects and keeps track of income 2. Manages day-to-day purchases, but needs help with banking, major purchases, etc 3. Incapable of handling money	- Can be scored dichotomously (0 = less able/dependent, 1 = more able/independent) - Or trichotomously (1 = unable/fully dependent, 2 = needs assistance, 3 = independent) - A higher (total) score reflects better performance or greater independence	- Internal consistency: people living with dementia: not available, CNCs: good - Reliability: adequate (Sikkes et al., 2008)	Allaire et al. (2009): - Used a shortened version of four items, including “ability to handle finances.” - Allowed for three responses; (1) “No, I never need help”; (2) “Yes, I have difficulty and need help”; (3) “Yes, I have difficulty but can do without help.” Mariani et al. (2008) Ogama et al. (2017) Tabira et al. (2020)
The Money Management Survey (MMS; Hoskin et al., 2005)	- Self and informant report instrument designed to assess (difficulties with) daily money management activities - Target population: people living with acquired brain injury - Consists of 11 concrete questions	**All 11 items relate to financial performance** Example items: **“Do they have problems using the ATM/filling out the forms?” (why?)** **“Do they pay the rent late?” (why?)** **“Have they ever been thrown out of their accommodation for not paying their rent?” (what happened?)**	- Items 1–4 are scored trichotomously (0 = never, 1 = sometimes, 2 = often), and allow for explanation (why?) - Items 5–6 are scored dichotomously (yes = 2, no = 0), and item 5 allows for explanation (what happened?) - Items 7–11 are scored trichotomously (0 = never, 1 = sometimes, 2 = often) - A lower (total) score reflects better performance	- Internal consistency: good (Cronbach’s *α* = 0.87) - Specificity: good for identifying money management problems of individuals with acquired brain injury (Hoskin et al., 2005)	Goverover et al. (2016) Goverover et al. (2019)
The Older Americans Resources and Services Activities of Daily Living scale (OARS ADL; Fillenbaum & Smyer, 1981)	- Self and informant report scale used to assess seven physical ADL and seven IADL - Target population: older adults	**“Money management”**	- Responses are scored on a 3-point scale ranging from dependent to independent - A lower score reflects better performance	- Internal consistency: poor (Cronbach’s *α* = 0.56) - Test–retest reliability: excellent (*r* = 0.91) - Interrater reliability: good (*r* = 0.80 or higher) - The OARS items were found to be “too easy,” as the instrument did not differentiate well between groups (Burholt et al., 2007; Doble & Fisher, 1998)	Tuokko et al. (2005): - Dichotomized the scores: people with “partially independent” and “dependent” scores were put in one group and compared to people with “fully independent” scores
The Seoul Instrumental Activities of Daily Living scale (S-IADL; Ku et al., 2004)	- Informant report instrument used to assess IADL - Target population: older adults in Korea - Developed for people living with AD - Consists of 15 items which have to do with complex ADL	**“Managing finances”**	- The scale differentiates between *actual* scores (based on observed functioning) and *potential* scores - Items are rated on a 4-point Likert scale - A lower score indicates better performance	- Internal consistency: excellent (Cronbach’s *α* = 0.94) - Test–retest reliability: excellent (*r* = 0.93) - Interrater reliability: moderate (κ = 0.55) - Sensitivity: good (83%) - Specificity: excellent (93%) (Ku et al., 2004)	Ahn et al. (2009) Cheon et al. (2015): - Instead of using the *potential* scores, Cheon et al. created a *cognitive score* where the informants were asked to imagine how the individual would perform the tasks if they did not have motor symptoms/dysfunction - Included a *physical score* calculated by subtracting the cognitive score from the actual score Kim et al. (2009)

*AD* Alzheimer’s disease, *ADL* activities of daily living, *AQ* Alzheimer’s questionnaire, *ATM* automated teller machine, *CFAR* current financial activities report, *CNCs* cognitively normal control participants, *Cronbach’s α* Cronbach’s alpha coefficient, *DAD* Disability Assessment for Dementia scale, *IADL* instrumental activities of daily living, *ICC* intraclass correlation, *κ* Kappa coefficient, *MCI* mild cognitive impairment, *MMSE* mini mental state examination, *n/a* not applicable, *OARS* Older Americans Resources and Services Activities of Daily Living scale, *r* correlation coefficient

^a^The third item of the “finance and correspondence” subscale does not address financial performance. However, as Charernboon and Lerthattasilp (2016) only reported on the total score of the “finance and correspondence” subscale, without differentiation between items, the domain score was considered for the present review

**Table 3:** Overview of studies on self or informant reported financial performance in people living with MCI (*k* = 12)

**First author (year), country**	**Study design**	**Sample characteristics**^**a**^		**Assessment of financial performance**	**Source**^**b**^	**Main outcomes**	**Conclusion**^**c**^
Ahn et al. (2009), South Korea	CS	**MCI** (*n* = 66) Age (y): 70.76 ± 7.33 Sex (m/f): 21/45 Education (y): 9.95 ± 5.14	MCI etiology: unknown MCI subtypes: - aMCI (*n* = 48) - naMCI (*n* = 18) CDR** (**stage): 0.5 (all participants) MMSE: 24.77 ± 3.10 GDS: n.r	Instrument: S-IADL Item: “managing finances”	Informant report	- No significant difference was observed between the reports for the MCI group and the reports of the CNC group on the “managing finances” item (*d* = 0.3)	Reported problems in “managing finances”: MCI = CNC
		**CNC** (*n* = 61) Age (y): 64.44 ± 5.60 Sex (m/f): 13/48 Education (y): 11.30 ± 4.10	CDR: n.r MMSE: 27.64 ± 1.44 GDS: n.r				
Allaire et al. (2009), USA	CS	**MCI** (*n* = 112) Age (y): 67.08 ± 9.57 Sex (m/f): 34%/66% Education (y): 12.30 ± 3.04	MCI etiology: unknown MCI subtypes: n.r CDR: n.r MMSE: 25.07 ± 3.16 GDS: n.r	Instrument: shortened version (4 items) of the Lawton – Brody IADL Item: “ability to handle finances”	Self-report	- A significantly greater proportion of MCI participants (i.e., 7%) reported dependency on others in the “ability to handle finances” as compared to the CNC group (i.e., 2%) (*d* = 0.2)	Self-reported problems in the “ability to handle finances”: MCI > CNC
		**CNC** (*n* = 399) Age (y): 68.97 ± 9.56 Sex (m/f): 22%/78% Education (y): 11.83 ± 2.65	CDR: n.r MMSE: 26.20 ± 3.01 GDS: n.r				
Charernboon and Lerthattasilp (2016), Thailand	CS^d^	**MCI** (*n* = 20) Age (y): 72.4 ± 7.9 Sex (m/f): 12/8 Education (y): 9.7 ± 4.9	MCI etiology: unknown MCI subtypes: n.r CDR (stage): 0.5 (all participants) MMSE: 26.3 ± 2.1 GDS: n.r	Instrument: DAD Category:^e^ “finance and correspondence”	Informant report	- Significantly lower scores were reported on the DAD category of “finance and correspondence” for the MCI participants as compared to the CNC group (*d* = 0.8), indicating a poorer functional status regarding financial performance in the MCI group - As compared to other (I)ADL categories, the category of “finance and correspondence” received the lowest score (indicating poorer functional status) in the MCI group and was the only category for which significant differences were observed between the MCI and CNC groups Associations: *When examining the correlation between MMSE scores, (I)ADL sub-scores, and stages of dementia in a combined sample of CNCs and participants with MCI, mild, moderate, or severe dementia, it was found that a MMSE score of 24 or lower (optimum cut-off) had a sensitivity of 0.84 and a specificity of 0.97 that people were unable to independently “organize their finances” (DAD item score; see *Table 2*), and that the “organization of finances” was the first ADL domain to be impaired along the stages of cognitive decline*	Reported problems in “finance and correspondence”: MCI > CNC
		**CNC** (*n* = 20) Age (y): 68.6 ± 5.2 Sex (m/f): 11/9 Education (y): 11.9 ± 5.0	CDR (stage): 0 (all participants) MMSE: 28.1 ± 2.4 GDS: n.r				
Gibson et al. (2020), USA	CS	**MCI** (*n* = 45) Age (y): 82.9 ± 9.4 Sex (m/f): 21/24 Education (y): 15.8 ± 0.5	MCI etiology: unknown MCI subtypes: n.r CDR (stage (mode)): 0.5 CDR (sum of boxes): 1.3 ± 1.4 MMSE: 26.6 ± 2.7 GDS (depression): 29.3%	Instrument: AMQoL Items: - “Do you want help with your finances?” - “Have others helped you with your finances?”	Self-report informant report^f^	- MCI participants were found to be significantly more likely to report that they want and have received help in their financial management as compared to the CNC group	Self-reported desired/received help in financial management: MCI > CNC
		**CNC** (*n* = 45) Age (y): 81.2 ± 1.0 Sex (m/f): 21/24 Education (y): 15.8 ± 0.4	CDR (stage (mode)): 0 CDR (sum of boxes): 0.1 ± 0.3 MMSE: 28.9 ± 1.6 GDS (depression): 11.4%				
Kenney et al. (2019), USA	CS^d,g^	**MCI** (*n* = 160) Dementia (*n* = 39) **CNC** (*n* = 71) Total sample (*n* = 270): Age (y): 71.87 ± 8.39 Sex (m/f): 100/170 Education (y): 14.13 ± 2.93	MCI etiology: unknown MCI subtypes: n.r CDR: n.r GDS: n.r CNCs were people seen at the hospital for a question of MCI or dementia but were classified as having normal cognition	Neuropsychologists evaluations of the “level of financial independence” in everyday functioning NB: If the neuropsychologist record indicated participants were never responsible for managing finances or this was unclear, participants were excluded from analysis	Informant report (clinician ratings): evaluations were based on various sources, including self and informant reports during clinical interviews, medical record reviews, and responses to clinically administered questionnaires	- Diagnoses and rated “levels of financial independence” were significantly related, but participants from each group (MCI, dementia, CNC) fell into each category of financial independence (independent, assisted, dependent) - 81.88% of the MCI participants were rated as independent in their financial management, as compared to 92.96% of the CNC group - 11.88% of the MCI participants were rated as being assisted by others (had some help/oversight) in managing their finances as compared to 4.23% of the CNC group - 6.25% of the MCI participants were rated as being fully dependent on others in their financial management as compared to 2.82% of the CNC group - 18.13% of the MCI participants utilized some level of assistance (either fully dependent/assisted) in their financial management, as compared to 7.05% of the CNCs group	MCI participants were more frequently rated as being assisted by or being dependent on others in their financial management as compared to the CNC group
Kim et al. (2009), South Korea	CS	**MCI** (*n* = 255) Age (y): 71.98 ± 6.0 Sex (m/f): 97/158 Education (y): 5.18 ± 4.7	MCI etiology: unknown ApoE e4-positive (*n* = 41) MCI subtypes: - sd-aMCI (*n* = 48) CDR: 0.40 ± 0.31 - md-aMCI (*n* = 109) CDR: 0.45 ± 0.19 - sd-aMCI (*n* = 66) CDR: 0.36 ± 0.25 - md-naMCI (*n* = 32) CDR: 0.30 ± 0.23 CDR (total MCI): 0.43 ± 0.25 MMSE (total MCI): 23.07 ± 4.53 GDS: (total MCI): 5.89 ± 4.06	Instrument: S-IADL Item: “managing finances”	Self-report	- Significantly higher scores were reported by the MCI participants (total MCI) as compared to the CNC group on the “managing finances” item (*d* = 0.2), indicating a poorer functional status regarding financial performance in the MCI group - When comparing the four MCI subtypes, significantly higher scores were reported by the md-aMCI participants as compared to the CNC group on the “managing finances” item, indicating a poorer functional status regarding financial performance in the md-aMCI group - No significant differences were observed between the other three MCI subtypes and the CNC group on the “managing finances” item Associations: *In the total MCI group (possibly combined with the CNC group), women scored significantly higher than men on the “managing finances” item, indicating a poorer functional status for women*	Self-reported problems in “managing finances”: MCI (total) > CNC md-aMCI > CNC sd-aMCI, sd-naMCI, md- naMCI = CNC
		**CNC** (*n* = 311) Age (y): 70.66 ± 5.95 Sex (m/f): 108/203 Education (y): 6.43 ± 4.78	ApoE e4-positive (*n* = 32) CDR: 0.26 ± 0.25 MMSE: 26.45 ± 3.28 GDS: 4.88 ± 3.91				

**First author (year), country**	**Study design**	**Sample characteristics**^**a**^		**Assessment of financial performance**	**Source**^**b**^	**Main outcomes**	**Conclusion**^**c**^
Malek-Ahmadi et al. (2012a), USA	CS	**aMCI** (*n* = 47) Age (y): 74.36 ± 7.19 Sex (m/f): 57%/43% Education (y): 14.43 ± 2.51	aMCI etiology: unknown aMCI subtypes: - sd-aMCI - md-aMCI CDR: n.r MMSE: 26.89 ± 1.90 GDS: n.r	Instrument: AQ Items: - “Excluding physical limitations, does the patient have trouble handling money?” - “Excluding physical limitations, does the patient have trouble paying bills or doing finances; OR are family members taking over finances because of concerns about ability?”	Informant report	- A significantly greater proportion of aMCI participants (i.e., 11%) were reported to have “trouble in handling money” as compared to the CNC group (i.e., 0%) (*d* = 0.5) - A significantly greater proportion of aMCI participants (i.e., 45%) were reported to have “trouble paying bills or doing finances” as compared to the CNC group (i.e., 6%) (*d* = 1.0) - The “trouble in handling money” item was no significant predictor for aMCI diagnosis - The “trouble in paying bills/doing finances” item was a strong, significant predictor for aMCI diagnosis	Reported problems in “handling money” and “paying bills or doing finances”: aMCI > CNC
		**CNC** (*n* = 51) Age (y): 78.59 ± 6.72 Sex (m/f): 43%/57% Education (y): 15.04 ± 3.03	CDR (stage): 0 (all participants) MMSE: 28.47 ± 1.27 GDS: n.r				
Mariani et al. (2008), Italy	CS	**aMCI** (*n* = 132) Age (y): 76.1 ± 5.8 Sex (m/f): 75/57 Education (y): 7.1 ± 4.2	aMCI etiology: unknown aMCI subtypes: n.r CDR (stage): 0.5 (all participants) MMSE: 25.7 ± 1.6 GDS: 3.9 ± 3.0	Instrument: Lawton – Brody IADL Item: “ability to handle finances”	Informant report	- A significantly greater proportion of aMCI participants (i.e., 9.1%) were reported to have impairments in the “ability to handle finances” as compared to CNC group (i.e., 2.8%) - This between-group difference remained significant after adjusting for age, gender, education, CIRS and GDS scores (*d* = 0.5) Associations: *- In both the aMCI and CNC groups (separate analyses), no association was found between each of the CIRS body system diseases and changes in the “ability to handle finances” item* *- In both the aMCI and CNC groups (separate analyses), performance on neuropsychological tests for episodic memory, language, attention/executive functioning and praxis was not significantly associated with reports on the “ability to handle finances” item* - *In CNCs no association was found between item specific IADL restriction and MMSE scores.*^*h*^	Reported problems in the “ability to handle finances”: aMCI > CNC
		**CNC** (*n* = 249) Age (y): 72.2 ± 7.5 Sex (m/f): 127/122 Education (y): 7.1 ± 4.0	CDR: n.r MMSE: 28.1 ± 1.2 GDS: 3.0 ± 2.7				
Schmitter-Edgecombe (2014), USA	CS^d^	**MCI** (*n* = 92) Age (y): 72.85 ± 8.16 Sex (m/f): 48.9%/51.1% Education (y): 15.23 ± 3.13	MCI etiology: unknown MCI subtypes: aMCI (*n* = 84) naMCI (*n* = 8) single-domain MCI (*n* = 35) multi-domain MCI (*n* = 57) CDR (stage): 0.5 (all participants) TICS: 32.51 ± 3.29 GDS: n.r	Instrument: IADL-C Category: “financial management”	Self-report^f^ Informant report	**-** Significant differences were observed between the informant reports for the MCI participants and the CNC group on all four “financial management” items - 29.3% of the MCI participants were reported not to “keep their financial records as well-organized as before” or needing (some) help with this task as compared to 7.1% of the CNC group - On the item “can manage a budget and business affairs” 23.7% of the MCI participants were reported not to perform this task as well as before, or needing (some) help with this task, as compared to 6.8% of the CNC group - On the item “balances a check book or credit card statement” 29.7% of the MCI participants were reported not to perform this task as well as before, or needing (some) help with this task, as compared to 4.1% of the CNC group - On the item “remembers whether bills were paid” 25.0% of the MCI participants were reported not to perform this task as well as before or needing (some) help with this task as compared to 3.6% of the CNC group - 13.4% of the MCI participants were reported to use a compensatory aid to keep their financial records organized, as compared to 9.5% of the CNC group - 17.1% of the MCI participants were reported to use a compensatory aid to manage a budget and business affairs, as compared to 5.6% of the CNC group - 14.9% of the MCI participants were reported to use a compensatory aid to balance check book or credit card statements as compared to 4.1% of the CNC group - 30.6% of the MCI participants were reported to use using a compensatory aid to remember whether bills were paid as compared to 24.6% of the CNC group	Reported problems in “financial management”: MCI > CNC
		**CNC** (*n* = 184) Age (y): 69.53 ± 10.93 Sex (m/f): 27.2%/72.8% Education (y): 16.51 ± 2.81	CDR (stage): 0 (all participants) TICS: 34.89 ± 2.66 GDS: n.r				
Shin et al. (2015), South Korea	CS	**aMCI** (*n* = 165) Age (y): 78.79 ± 8.99 Sex (m/f): 55/110 Education (y): 5.30 ± 5.31	aMCI etiology: unknown aMCI subtypes: n.r CDR: n.r MMSE: 19.94 ± 5.50 GDS: 12.53 ± 6.98	Instrument: K-IADL Item: “handling money”	Self-report	- Significantly higher scores were reported by the aMCI participants compared to the CNC (*d* = 0.6) and the SCI (*d* = 0.5) groups on the “handling money” item, indicating a poorer functional status regarding financial performance in the aMCI group - Significantly higher scores were reported by the naMCI group compared to the CNC (*d* = 0.6) and the SCI (*d* = 0.4) groups on the “handling money” item, indicating a poorer functional status regarding financial performance in the naMCI group - No significant differences were observed between the SCI and CNC groups on the “handling money” item - After controlling for age, gender, educational level, GDS, and the POMA subscales, only the difference between the aMCI and SCI groups remained significant, with scores indicating a poorer functional status for the aMCI than for the SCI group	Self-reported problems in “handling money”: SCI = CNC aMCI > SCI, CNC na-MCI > SCI, CNC Self-reported problems in “handling money” after controlling for covariates: aMCI > SCI
		**naMCI** (*n* = 98) Age (y): 79.52 ± 8.77 Sex (m/f): 41/57 Education (y): 6.10 ± 5.19	naMCI etiology: unknown naMCI subtypes: n.r CDR: n.r MMSE: 21.87 ± 4.21 GDS: 12.36 ± 6.15				
		**SCI** (*n* = 107) Age (y): 73.71 ± 7.43 Sex (m/f): 43/64 Education (y): 6.73 ± 5.76	CDR: n.r MMSE: 23.67 ± 3.9 GDS: 12.12 ± 6.69 SCI participants had subjective cognitive complaints, no dementia and intact functional activity, but no objective cognitive impairments				
		**CNC** (*n* = 332) Age (y): 73.39 ± 7.38 Sex (m/f): 182/150 Education (y): 9.74 ± 5.32	CDR: n.r MMSE: 25.70 ± 2.93 GDS: 8.85 ± 6.33				
Terada et al. (2019), Japan	CS^d^	**MCI** (*n* = 199) Age (y): 77.0 ± 7.0 Sex (m/f): 75/126 Education: n.r	MCI etiology: unknown MCI subtypes: sd-aMCI (*n* = 149) md-aMCI (*n* = 36) naMCI (*n* = 14) CDR: n.r HDS-R: 23.2 ± 4.0 GDS: n.r	Frequency of occurrence of certain problems, including: - “massive, recurrent buying” - “trouble with wealth management” - “trouble with money management”	Informant reported: the frequency of each problem was evaluated by experts in clinical medicine for dementia or trained psychologists and was based on information from family caregivers	- MCI participants were reported to “cause trouble” significantly more frequently as compared to the CNC group in the fields of “massive, recurrent buying”, “trouble with wealth management” and “trouble with money management” - 12.6% of the MCI participants were reported to “cause trouble” in the field of “massive, recurrent buying” once a year or more, and 10.1% once a month or more, as compared to 0% of the CNC group - 9.5% of the MCI participants were reported to “cause trouble with wealth management” once a year or more, and 7.5% once a month or more, as compared to 0% of the CNC group - 8.0% of the MCI participants were reported to “cause trouble with money management” once a year or more, and 6.5% once a month or more, as compared to 0% of the CNC group	Reported problems in financial performance regarding “massive, recurrent buying,” “trouble with wealth management,” and “trouble with money management”: MCI > CNC
		**CNC** (*n* = 60) Age (y): 73.7 ± 7.7 Sex (m/f): 22/39 Education: n.r	CDR: n.r HDS-R: 28.1 ± 1.7 GDS: n.r				
Tuokko et al. (2005), Canada	CS and longitudinal: participants were assessed at two time points (T1 and T2), with a five-year time interval T1: 937 participants were included, 225 were diagnosed with MCI and 712 were classified as CNC T2: Five years later, all participants were approached again and those who agreed to participate once more received a clinical evaluation. At T2, 101 participants were diagnosed with MCI, 95 with dementia^d^ and 166 were classified as CNC	Total sample: T1 (*n* = 937) T2 (*n* = 362) Age: participants were all > 65 years at T1(inclusion) Sex: at both time points there were roughly half as many males as females	n.r	Instrument: OARS Item: “money management”	Self-report	- T1: a significantly greater proportion of MCI participants (i.e., 23.3%) reported to be (partially) dependent in their money management as compared to the CNC group (i.e., 6.1%) (*d* = 0.5) - T2: of the participants who showed no functional impairment in their money management at T1, significantly more MCI participants (i.e., 37.5%) than CNCs (i.e., 5.8%) did report to be (partially) dependent in their money management after five years (*d* = 0.6) NB: given the small sample of MCI participants in this analysis (*n* = 8), the statistical comparisons must be viewed with caution Associations: *- Regression analysis in MCI and CNC participants at T1 showed that performance on seven neuropsychological tests in four domains (memory, verbal abilities, visuo-constructional ability, and attention and processing speed) could significantly predict money management ability, and adding demographic variables (age, sex, education) to the analysis showed females to be significantly more likely to have difficulty in money management than males* *- Performance on these seven neuropsychological tests contributed to the prediction of future difficulty in money management, and persons with poorer memory at T1 were more likely to have difficulty in money management at T2*	Reported dependency in “money management” T1: MCI > CNC Reported problems in “money management” at T2 of people being ‘independent’ at T1: MCI > CNC
		**T1 – MCI** (*n* = 225) Age: 80.76 (SD n.r.) Sex: n.r Education (y): 8.15 (SD n.r.)	MCI etiology: unknown MCI subtypes: n.r CDR: n.r Modified MMSE (3MS): 70.92 (SD n.r.) GDS: n.r				
		**T1 – CNC** (*n* = 712) Age: 78.44 (SD n.r.) Sex: n.r Education (y): 8.88 (SD n.r.)	MCI etiology: unknown MCI subtypes: n.r CDR: n.r Modified MMSE (3MS): 83.02 (SD n.r.) GDS: n.r				
		**T2 – MCI** (*n* = 101) Descriptive statistics: n.r	n.r				
		**T2 – CNC** (*n* = 166) Descriptive statistics: n.r	n.r				

*aMCI* amnestic mild cognitive impairment, *AMQoL* aging and memory quality of life, *ApoE* apolipoprotein E, *AQ* Alzheimer’s questionnaire, *CDR* Clinical Dementia Rating, *CIRS* cumulative illness rating scales, *CNC* cognitively normal control (group), *CS* cross-sectional design, *d* Cohen’s *d* (effect sizes were calculated and converted making use of www.psychometrica.de), *DAD* Disability Assessment for Dementia, *GDS* Geriatric Depression Scale, *HDS-R* Hasegawa dementia rating scale – revised, *(I)ADL* (instrumental) activities of daily living, *IADL-C* Instrumental Activities of Daily Living – Compensation, *Lawton – Brody IADL* Lawton – Brody Instrument Activities of Daily Living scale, *k* number of studies, *K-IADL* Korean Instrumental Activities of Daily Living scale, *MCI* mild cognitive impairment, *md-aMCI* multiple-domain amnestic mild cognitive impairment, *md-naMCI* multiple-domain non-amnestic mild cognitive impairment, *m/f* male/female ratio, *MMSE* mini mental state examination;* n* sample size, *naMCI*. non-amnestic mild cognitive impairment, *NB* nota bene, *n.r.* not reported, *OARS* Older Americans Resources and Services Scale, *SCI* subjective cognitive impairment, *SD* standard deviation, *sd-aMCI* single-domain amnestic mild cognitive impairment, *sd-naMCI* single-domain non-amnestic mild cognitive impairment, *S-IADL* Seoul-Instrumental Activities of Daily Living scale, *TICS* Telephone Interview for Cognitive Status, *T1* time point one, *T2* time point two, *USA* United States of America, *y* number of years

^a^All descriptive variables are reported as mean ± standard deviation, unless otherwise indicated

^b^Refers to the source (self or informant report) of the relevant patient group(s) only, unless otherwise specified

^c^ > and < refer to significant differences only, based on the alpha levels used in the original studies

^d^Study also included one or more dementia group(s) with mixed/unspecified etiologies

^e^The item category “finance and correspondence” consists of four items (see Table 2), one of which does not address financial performance, but was still considered for review as part of the item category

^f^No financial performance scores were reported for the self or informant report version of the assessment

^g^Retrospective chart review of outpatients of a hospital’s neuropsychology program

^h^In the aMCI group, the association between MMSE scores and IADL items is not (clearly) reported on, and data are not shown for item specific analyses

**Table 4:** Overview of studies on self or informant reported financial performance in people living with (mild) AD (*k* = 1)

**First author (year), country**	**Study design**	**Sample characteristics**^**a**^		**Assessment of financial performance**	**Source**^**b**^	**Main outcomes**	**Conclusion**^**c**^
Tabira et al. (2020), Japan	CS NB: to examine age-related changes in (I)ADL, participants of both groups (very mild AD and CNC) were divided into age groups of 3-year intervals for part of the analyses: 65–67 (*n* = 8, *n* = 8); 68–70 (*n* = 8, *n* = 8); 71–73 (*n* = 4, *n* = 4); 74–76 (*n* = 14, *n* = 14); 77–79 (*n* = 23, *n* = 23); 80–82 (*n* = 26, *n* = 26); 83–85 (*n* = 14, *n* = 14); 86–88 (*n* = 10, *n* = 10)	*Before matching:* **Very mild AD** (n = 107) Age (y): 78.1 ± 6.0 Sex (m/f): 49/58 Education (y): 11.6 ± 2.7 *After matching (for age, sex):* **Very mild AD** (n = 104) Age (y): 77.9 ± 5.9 Sex (m/f): 47/57 Education (y): 11.6 ± 2.7	CDR: n.r *Before matching:* MMSE: 25.4 ± 1.5 *After matching (for age, sex):* MMSE: 25.6 ± 1.5 GDS: n.r	Instrument: Lawton – Brody IADL Item: “ability to handle finances”	Informant report	- A lower reported independence in the “ability to handle finances” was significantly associated with a greater odds of being in the very mild AD group (before matching) (*d* = 2.2) **Comparison between age groups (after matching):** - The independence ratio for the “ability to handle finances” was significantly lower for very mild AD participants than for CNCs (all age groups combined) - The independence ratios for the “ability to handle finances” were significantly lower for very mild AD participants than for CNCs in all individual age groups - Both groups (very mild AD and CNC) showed decreases in (I)ADL with age; however, the decreasing slope and items on which decline was observed differed between the very mild AD and CNC group - For the very mild AD participants, the decrease in independence in the “ability to handle finances” began in a younger age group (first decline relative to younger age group; 68–70) as compared to CNCs (first decline relative to younger age group; 80–82), and the decreasing slope for the “ability to handle finances” across age was steeper in very mild AD participants than in CNCs	Reported dependence in the “ability to handle finances” very mild AD > CNC Independence in the “ability to handle finances” decreased with age in very mild AD and CNC groups (Rate of) decrease with age: very mild AD > CNC
		*Before matching:* **CNC** (n = 682) Age (y): 73.7 ± 5.5 Sex (m/f): 307/375 Education (y): 11.0 ± 3.8 *After matching (for age, sex):* **CNC** (*n* = 104) Age (y): 77.9 ± 5.9 Sex (m/f): 47/57 Education (y): 11.2 ± 3.3	CDR: n.r *Before matching:* MMSE: 28.0 ± 1.9 *After matching (for age, sex):* MMSE: 26.0 ± 1.9 GDS: n.r				

*AD* Alzheimer’s disease, *CDR* Clinical Dementia Rating, *CNC* cognitively normal control (group), *CS* cross-sectional design, *d* Cohen’s *d* (effect sizes were calculated and converted making use of www.psychometrica.de), *GDS* Geriatric Depression Scale *(I)ADL* (instrumental) activities of daily living, *k* number of studies, *m/f* male/female ratio, *MMSE* mini mental state examination, *n* sample size, *NB* nota bene, *n.r.* not reported, *y* number of years

^a^All descriptive variables are reported as mean ± standard deviation, unless otherwise indicated.

^b^Refers to the source (self or informant report) of the relevant patient group(s) only, unless otherwise specified.

^c^ > and < refer to significant differences only, based on the alpha levels used in the original studies

**Table 5:** Overview of studies on self or informant reported financial performance including an MCI and a (mild) AD group (*k* = 6)

**First author (year), country**	**Study design**	**Sample characteristics**^**a**^		**Assessment of financial performance**	**Source**^**b**^	**Main outcomes**	**Conclusion**^**c**^
Brown et al. (2011), USA	CS	**aMCI** (*n* = 394) Age (y): 74.86 ± 7.40 Sex (m/f): 256/138 Education (y): 15.65 ± 3.04	aMCI etiology: unknown aMCI subtypes: - sd-aMCI - md-aMCI CDR (stage): 0.5 (all participants) CDR (sum of boxes): 1.6 ± 0.89 MMSE: 27.04 ± 1.78 GDS (total score): < 6 (no significant depression)	Instrument: FAQ Items: - “writing checks, paying bills, balancing check book” - “assembling tax records, business affairs, or papers”	aMCI/mild AD: informant report CNC: self-report	- On the item “writing checks, paying bills, or balancing check book” 33.8% of the aMCI participants and 88.1% of the mild AD participants were reported to have deficits, as compared to 2.2% of the CNC group reporting deficits on this item - On the item “assembling tax records, business affairs, or papers” 42.9% of the aMCI participants and 91.2% of the mild AD participants were reported to have deficits, as compared to 1.7% of the CNC group reporting deficits on this item - The frequency of reported deficits on the two financial performance items increased markedly from CNCs to aMCI participants to participants with mild AD (Cochrane-Armitage linear trends test)	The informants of the mild AD group reported more problems on the financial performance items than the informants of the aMCI group, who reported more problems than the CNCs
		**Mild AD** (*n* = 193) Age (y): 75.33 ± 7.48 Sex (m/f): 102/91 Education (y): 14.71 ± 3.13	CDR (stage): 0.5 or 1 (all participants) CDR sum of boxes: 4.3 ± 1.64 MMSE: 23.34 ± 2.06 GDS (total score): < 6 (no significant depression)				
		**CNC** (*n* = 229) Age (y): 75.9 ± 5.00 Sex (m/f): 119/110 Education (y): 16.04 ± 2.9	CDR (stage) = 0 (all participants) CDR (sum of boxes): 0.03 ± 0.12 MMSE: 29.11 ± 1.00 GDS (total score): < 6 (no significant depression)				
Gerstenecker et al. (2019), USA	CS	**aMCI** (*n* = 65) Age (y): 72.6 ± 7.4 Sex (m/f): 32/33 Education (y): 15.2 ± 2.6	aMCI etiology: unknown aMCI subtypes: n.r CDR: n.r MMSE: 27.4 ± 3.2 GDS: n.r	CFCF^d^	Self-report Informant report	**Self-reports:** - No significant difference was observed between the self-reported “global financial capacity” of the aMCI participants and the CNC group - The mild AD participants reported a significantly lower “global financial capacity” as compared to the aMCI and CNC groups, indicating a worse overall financial performance in the mild AD group - The self-reports of the aMCI participants were significantly worse as compared to the CNC group for the domains of “bank statement management” and “bill payment” and did not differ significantly for the other domains - The self-reports of the mild AD participants were significantly worse as compared to the CNC group for all domains of the CFCF, except for “basic monetary skills” and “financial judgment”, for these domains, self-reports did not differ significantly between groups - The self-reports of the mild AD participants were significantly worse as compared to the aMCI group for the domains of “check book management,” “bank statement management,” and “knowledge of assets/estate,” and did not differ significantly for the other domains - Comparisons across groups (aMCI, mild AD, CNC): global financial capacity: *d* = 0.9, basic monetary skills: *d* = 0.5, financial conceptual knowledge: *d* = 0.6, cash transactions: *d* = 0.5, checkbook management: *d* = 0.6, bank statement management: *d* = 0.8, financial judgment: *d* = 0.3, bill payment: *d* = 0.8, knowledge of assets/estate: *d* = 0.5, investment decisions: *d* = 0.5 Associations: *No associations were found between the MMSE and CFCF self-reports in any of the study groups (aMCI, mild AD and CNC)* **Informant reports:** - The aMCI and mild AD participants were reported to have a significantly lower “global financial capacity” as compared to the CNC group, indicating a worse overall financial performance in the aMCI and mild AD groups - The mild AD participants were reported to have a significantly lower “global financial capacity” as compared to the aMCI group, indicating a worse overall financial performance in the mild AD group - The informant reports for the aMCI participants were significantly worse as compared to the CNC group for the domains of “cash transactions,” “check book management,” “bank statement management,” “financial judgment,” “bill payment,” and “investment decisions.” - The informant reports for the mild AD participants were significantly worse as compared to the CNC group for all domains of the CFCF - The informant reports for the mild AD participants were significantly worse as compared to the aMCI group for all domains of the CFCF, except for “investment decisions” for this domain, informant reports did not differ significantly between groups - Comparisons across groups (aMCI, mild AD, CNC): global financial capacity: *d* = 1.6, basic monetary skills:* d* = 0.9, financial conceptual knowledge: *d* = 1.2, cash transactions: *d* = 1.3, checkbook management: *d* = 1.5, bank statement management: *d* = 1.5, financial judgment: *d* = 1.3, bill payment: *d* = 1.8, knowledge of assets/estate: *d* = 0.9, investment decisions: *d* = 1.1 Associations: *No associations were found between the MMSE and CFCF*^*3*^* informant reports in any of the study groups (aMCI, mild AD and CNC)*	Self-reported problems in financial performance: Global financial capacity: aMCI = CNC Mild AD > aMCI, CNC Domain level: Mild AD > aMCI > CNC Informant reported problems in financial performance: Global financial capacity: Mild AD > aMCI > CNC Domain level: Mild AD > aMCI > CNC
		**Mild AD** (*n* = 59) Age (y): 74.5 ± 8.7 Sex (m/f): 32/27 Education (y): 14.5 ± 3.2	CDR: n.r MMSE: 23.7 ± 3.1 GDS: n.r				
		**CNC** (*n* = 64) Age (y): 70.6 ± 7.4 Sex (m/f): 19/45 Education (y): 15.8 ± 2.3	CDR: n.r MMSE: 28.7 ± 3.9 GDS: n.r				
Griffith et al. (2003), USA	CS	**aMCI** (*n* = 21) Age (y): 68.1 ± 8.8 Sex (m/f): 10/11 Education (y): 14.3 ± 2.2	aMCI etiology: unknown aMCI subtypes: n.r CDR (stage): 0 = 9.5% 0.5 = 90.5% MMSE: 28.4 ± 1.2 GDS: 5.3 ± 4.6	CFCF	Informant report	- The aMCI participants were reported to have a significantly lower “global financial capacity” as compared to the CNC group (i.e., the aMCI group had more “needs assistance” ratings than the CNCs), indicating a worse overall financial performance in the aMCI group - The mild AD participants were reported to have a significantly lower “global financial capacity” as compared to the aMCI or CNC group, indicating a worse overall financial performance in the mild AD group - No significant differences were observed between the aMCI participants and the CNC group on the domain-level of the CFCF - The informant reports for the mild AD participants were significantly worse as compared to the aMCI or CNC group for the domains of “conceptual knowledge,” “cash transactions,” “check book management,” “bank statement management,” “financial judgment,” and “bill payment,” no significant between-group differences were observed for the domains of “basic money skills” and “knowledge of assets/estate.” - In the informant reports, CNCs had functional limitations (i.e., ratings of “needs assistance” or “incapacity”) in 5% of the cases, aMCI participants in 13% of the cases and mild AD participants in 49% of the cases	Reported problems in financial performance: Global financial capacity: aMCI > CNC Mild AD > aMCI or CNC Domain level: aMCI = CNC Mild AD > aMCI or CNC
		**Mild AD** (*n* = 22) Age (y): 71.5 ± 9.2 Sex (m/f): 9/13 Education (y): 14.5 ± 2.5	CDR (stage): 1 (all participants) MMSE: 24.1 ± 2.6 GDS: 8.2 ± 6.0				
		**CNC** (*n* = 21) Age (y): 66.7 ± 7.2 Sex (m/f): 7/14 Education (y): 14.3 ± 2.7	CDR (stage): 0 = 95.2% 0.5 = 4.8% MMSE: 29.3 ± 1.0 GDS: 3.1 ± 2.6				
Lui et al. (2013), Hong Kong	CS	**aMCI** (*n* = 92) Age (y): 77.8 ± 6.8 Sex (m/f): 26/66 Education (y): 3.0 ± 3.2	aMCI etiology: unknown aMCI subtypes: n.r CDR (stage): > 0 and < 1 (all participants) MMSE: 25.3 ± 2.6 GDS: n.r	- Clinician ratings on whether an individual is “mentally competent” or “mentally incompetent” to make daily financial management decisions (e.g., paying bills), based on patient interviews - The clinical judgment was based on the criteria in Sect. 3 of the Mental Capacity Act 2005 of the United Kingdom - Clinicians (geriatric psychiatrists) were blinded for diagnosis and scores on a performance-based financial capacity test	Informant report (clinician ratings)	- Based on clinician ratings, 94.6% of the aMCI participants were found to be “mentally competent” to make financial decisions as compared to 97.98% of the CNC group (no significant between-group difference) (*d* = 0.2) - 53.3% of the mild AD participants were found to be “mentally competent” to make financial decisions, which was significantly less than the percentage of “mentally competent” participants in the aMCI and CNC groups (mild AD compared to aMCI, *d* = 1.1) - Associations: *No significant difference in gender ratio was found between “mentally competent” and “mentally incompetent participants.”*	Clinician ratings of “mental incompetence to make financial decisions”: aMCI = CNC mild AD > aMCI, CNC
		**Mild AD** (*n* = 90) Age (y): 82.2 ± 6.6 Sex (m/f): 14/76 Education (y): 1.7 ± 3.3	CDR (stage): ≥ 1 and < 2 (all participants) MMSE: 19.7 ± 2.5 GDS: n.r				
		**CNC** (*n* = 93) Age (y): 74.2 ± 6.5 Sex (m/f): 10/83 Education (y): 4.3 ± 3.7	CDR (stage): 0 (all participants) MMSE: 26.6 ± 2.5 GDS: n.r				
Ogama et al. (2017), Japan	CS	**aMCI** (*n* = 44) Age (y): 75.1 ± 5.6 Sex: all female Education (y): 10.8 ± 2.0	aMCI etiology: unknown aMCI subtypes: n.r CDR: n.r MMSE: 24.8 ± 2.6 GDS: 3.8 ± 2.5	Instrument: Lawton – Brody IADL Item: “ability to handle finances”	Unknown	- No significant difference was observed on the “ability to handle finances” item between the aMCI and CNC groups (*d* = 0) - A significantly lower score on the “ability to handle finances” item was reported in the AD group as compared to the aMCI (*d* = 0.4) and CNC (*d* = 0.4) groups, indicating a poorer functional status regarding financial performance in the AD group To assess the difference in IADL subdomains between people with different cognitive statuses, AD participants were subclassified into three groups by total MMSE score (AD 24–30, AD 20–23, and AD 15–19): - As compared to the CNC group, a significantly lower score on the “ability to handle finances” item was reported in the AD 20–23 and AD 15–19 groups, but not in AD 24–30 or aMCI groups, indicating the “ability to handle finances” decreased significantly with increasing cognitive impairment Associations: *Multiple logistic regression analysis was performed in a combined group of aMCI and AD participants* *- Model 1: age, WMH in the frontal lobe, brain parenchyma, scores on the ADAS, scores on the vitality index, and grip strength were entered as ‘potential independent risk factors for impairment’ on the financial performance item. Only WMH in the frontal lobe was found to be significantly associated with the “ability to handle finances”* *- Model 2: MMSE, GDS, and timed up and go scores were added to the previous model (model 1) as ‘potential risk factors’. Only MMSE scores were found to be significantly associated with the “ability to handle finances”*	Reported problems in the “ability to handle finances”: aMCI = CNC AD > aMCI, CNC AD subgroups: AD MMSE 24–30 = CNC AD MMSE 20–23 > CNC AD MMSE 15–19 > CNC
		**AD** (*n* = 227) Age (y): 77.8 ± 4.9 Sex: all female Education (y): 9.9 ± 2.0	CDR: n.r MMSE: 20.4 ± 3.5 GDS: 4.4 ± 2.6				
		**CNC** (*n* = 76) Age (y): 73.1 ± 4.7 Sex: all female Education (y): 11.3 ± 2.3	CDR: n.r MMSE: 28.4 ± 1.9 GDS: 4.6 ± 3.1 CNCs visited the hospital for suspected memory disorder but were evaluated as having a normal cognition				
Yeh et al. (2011), Taiwan	CS	**sd-aMCI** (*n* = 56) Age (y): 77.5 ± 6.7 Sex (m/f): 71.4%/28.6% Education (y): 12.6 ± 3.6	aMCI etiology: unknown CDR (stage): < 1 (all participants) MMSE: 26.6 ± 1.6 GDS: 3.8 ± 3.2	Instrument: C-DAD Item:^e^ - “Organize his/her finances to pay his/her bills”	Informant report	- A significantly greater proportion of sd-aMCI participants (i.e., 14%), md-aMCI participants (i.e., 27%), and mild AD participants (i.e., 82%) were reported to present with functional deficits in the “organization of finances to pay bills” as compared to the CNC group (2%) - A significantly greater proportion of mild AD participants (i.e., 82%) were reported to present with functional deficits in the “organization of finances to pay bills” as compared to the sd-aMCI and md-aMCI groups - No significant differences were observed between the sd-aMCI and md-aMCI groups in the reported “organization of finances to pay bills”	Reported problems in the “organization of finances to pay bills”: sd-aMCI > CNC md-aMCI > CNC sd-aMCI = md-aMCI mild AD > CNC mild AD > sd-aMCI, md-aMCI
		**md-aMCI** (*n* = 94) Age (y): 78.9 ± 5.8 Sex (m/f): 60.6%/39.4% Education (y): 10.5 ± 3.5	aMCI etiology: unknown CDR (stage): < 1 (all participants) MMSE: 25.8 ± 1.6 GDS: 4.5 ± 3.4				
		**Mild AD** (*n* = 102) Age (y): 79.6 ± 6.1 Sex (m/f): 61.8%/38.2% Education (y): 11.2 ± 3.5	CDR (stage): 1 (all participants) MMSE: 20.9 ± 3.1 GDS: 3.6 ± 2.7				
		**CNC** (*n* = 64) Age (y): 76.5 ± 6.6 Sex (m/f): 62.5%/37.5% Education (y): 13.3 ± 3.4	CDR (stage): n.r MMSE: 28.5 ± 1.3 GDS: 3.6 ± 2.9				

*ADAS* Alzheimer Disease Assessment Scale, *aMCI* amnestic mild cognitive impairment, *AD* Alzheimer’s disease, *C-DAD* Disability Assessment for Dementia Chinese version, *CDR* Clinical Dementia Rating, *CFCF* Current Financial Capacity Form, *CNC* cognitively normal control (group), *CS* cross-sectional design, *d* Cohen’s *d* (effect sizes were calculated and converted making use of www.psychometrica.de), *FAQ* Functional Activities Questionnaire, *GDS* Geriatric Depression Scale, *IADL* instrumental activities of daily living, *k* number of studies, *Lawton – Brody IADL,* Lawton – Brody Instrument Activities of Daily Living scale, *MCI* mild cognitive impairment, *md-aMCI* multiple-domain amnestic mild cognitive impairment, *m/f* male/female ratio, *MMSE* mini mental state examination,* n* sample size, *NB* nota bene, *n.r.* not reported, *sd-aMCI* single-domain amnestic mild cognitive impairment, *USA* United States of America, *WMH* white matter hyperintensity, *y* number of years

^a^All descriptive variables are reported as mean ± standard deviation, unless otherwise indicated

^b^Refers to the source (self or informant report) of the relevant patient group(s) only, unless otherwise specified

^c^ > and < refer to significant differences only, based on the alpha levels used in the original studies

^d^The authors refer to the CFCF as the current financial activities report (CFAR) in this study (see Table 2)

^e^Presumably, the two other C-DAD items on financial performance (Table 2) were also used in this study. As the authors only describe the “main different IADL items among study participants,” it remains unclear, however, whether (significant) between-group differences were observed for these two items as well.

**Table 6:** Overview of studies on self or informant reported financial performance in people living with PD (*k* = 1)

**First author (year), country**	**Study design**	**Sample characteristics**^**a**^		**Assessment of financial performance**	**Source**^**b**^	**Main outcomes**	**Conclusion**^**c**^
Cheon et al. (2015), South Korea	CS	**PD** (*n* = 48) Age (y): 68.7 ± 7.1 Sex (m/f): 23/25 Education (y): 6.9 ± 4.7	Symptom duration (y): 6.8 ± 4.6 UPDRS (motor sub-score): 33.0 ± 9.1 H&Y stage: 2.4 ± 0.7 CDR: n.r MMSE: 27.2 ± 2.7 GDS: n.r	Instrument: Modified^d^ S-IADL Item: “managing finances”	PD/PDD/AD: Informant report NC: Self-report	**PD compared to NC:** - No significant difference was observed between the actual scores (*d* = 0.3) and cognitive scores (*d* = 0) of the PD participants as compared to the NC group on the “managing finances” item - In the PD group, the cognitive scores were significantly lower (indicating better functioning) than the actual scores on the “managing finances” item - In the NC group, no significant difference was observed between the cognitive scores and the actual scores on the “managing finances” item **PDD compared to PD and NC:** - Significantly higher actual scores and cognitive scores were reported for the PDD participants as compared to the PD (*d* = 0.7, *d* = 0.7) and NC (*d* = 1, *d* = 0.6) groups on the “managing finances” item, indicating a poorer functional status regarding financial performance in the PDD group - In the PDD group, no significant difference was observed between the cognitive scores and the actual scores on the “managing finances” item **AD compared to PDD, PD, and NC:** - No significant difference was observed between the actual scores of the PDD and AD participants (*d* = 0.3), significantly higher scores were reported for both groups as compared to the PD and NC groups, indicating a poorer functional status regarding financial performance in the PDD and AD groups (AD compared to NC, *d* = 1.5) - Significantly higher cognitive scores were reported for the AD group as compared to the PDD (*d* = 0.8), PD, and NC groups, indicating a poorer functional status regarding financial performance in the AD group (AD compared to NC, *d* = 1.5) - In the AD group, no significant difference was observed between the cognitive scores and the actual scores on the “managing finances” item	Reported problems in “managing finances”: PD = NC PDD > PD, NC AD > PD, NC Actual scores: AD = PDD Cognitive scores: AD > PDD Within-group differences in “managing finances”: PD: cognitive scores < actual scores NC, PDD, AD: cognitive scores = actual scores
		**PDD** (*n* = 24) Age (y): 70.4 ± 6.3 Sex (m/f): 8/16 Education (y): 4.3 ± 5.2	Symptom duration (y): 8.0 ± 5.2 UPDRS (motor sub-score): 34.3 ± 8.2 H&Y stage: 2.8 ± 0.8 CDR: n.r MMSE: 18.1 ± 4.2 GDS: n.r				
		**AD** (*n* = 24) Age (y): 71.0 ± 8.6 Sex (m/f): 9/15 Education (y): 6.8 ± 5.8	Symptom duration (y): 6.9 ± 4.1 CDR: n.r MMSE: 16.7 ± 5.7 GDS: n.r				
		**NC** (*n* = 25) Age (y): 68.6 ± 8.2 Sex (m/f): 10/15 Education (y): 7.2 ± 4.7	CDR: n.r MMSE: 27.3 ± 3.3 GDS: n.r				

*AD* Alzheimer’s disease, *CDR* Clinical Dementia Rating, *CS* cross-sectional design, *d* Cohen’s *d* (effect sizes were calculated and converted making use of www.psychometrica.de), *GDS* Geriatric Depression Scale, *H&Y stage* Hoehn & Yahr staging, *k* number of studies, *m/f* male/female ratio, *MMSE* mini mental state examination, *n* sample size, *NC* normal control (group), *n.r.* not reported, *PD* Parkinson’s disease, *PDD* Parkinson’s disease dementia, *S-IADL scale* Seoul-Instrumental Activity of Daily Living scale, *UPDRS* Unified Parkinson’s disease rating scale (motor examination), *y* number of years

^a^All descriptive variables are reported as mean ± standard deviation, unless otherwise indicated

^b^Refers to the source (self or informant report) of the relevant patient group(s) only, unless otherwise specified

^c^ > and < refer to significant differences only, based on the alpha levels used in the original studies

^d^Modifications to the instrument are explained in Table 2

**Table 7:** Overview of studies on self or informant reported financial performance in people living with MS (*k* = 2)

**First author (year), country**	**Study design**	**Sample characteristics**^**a**^		**Assessment of financial performance**	**Source**^b^	**Main outcomes**	**Conclusion**^**c**^
Goverover et al. (2016), USA	CS	**MS** (*n* = 30) Age (y): 47.9 ± 10.6 Sex (m/f): 7/23 Education (y): 16.3 ± 2.0	MS subtypes: - RR (60%) - PP (26.7%) - SP (13.3%) Disease duration (mo): 95.9 ± 53.3 MSFC (z-score): − 1.29 ± 2.01 Global cognition: n.r CMDI (total score): 54.9 ± 14.6	MMS	Self-report	- Significantly more problems in money management were reported by the MS participants as compared to the NC group on the MMS (total MMS score) (*d* = 0.6) - Of the 10 MMS items used, the likelihood of increased problems was significantly greater in the MS group with regard to “operating an ATM” (i.e., 13.3%), “owning debt for bills they have not paid” (i.e., 23.3%), and “needing to borrow money” (i.e., 30%) as compared to the NC group (i.e., 0%, 4.3%, and 8.7%, respectively). No significant between-group differences were observed in the likelihood for the other 7 items - Associations: *In the MS group (possibly combined with the NC group), self-reported problems in managing money were significantly correlated with a higher reported dysfunction on an IADL scale and on a functional status questionnaire for everyday life task performance, social interactions and problem solving (FBP), but not with affect symptomatology (depression and anxiety ratings) or performance-based neuropsychological test scores in the domains of learning and memory, executive functions, and processing speed and working memory*	Self-reported problems in money management: MS > NC
		**NC** (*n* = 23) Age (y): 50 ± 9.4 Sex (m/f): 10/13 Education (y): 16 ± 2.3	MSFC (z-score): 0.47 ± 0.35 Global cognition: n.r CMDI (total score): 45.6 ± 8.3				
Goverover et al. (2019), USA	CS NB: Scores of the money management portion of a performance-based test (AR) and both versions of the MMS (self and informant report) were summed and averaged for the MS participants (observed range: 0.33–9), from which a cut-off of 3 points was derived that was used to divide the MS participant sample into two groups: efficient money management (score ≤ 3), and inefficient money management (score > 3)	**MS efficient money management** (*n* = 34) Age (y): 51.6 ± 9.3 Sex (m/f): 4/30 Education (y): 15.8 ± 1.9	MS subtypes: - RR (95.7%) - PP (0%) - SP (4.3%) Disease duration (mo): 198.4 ± 104.8 MSFC (z-score): − 0.05 ± 0.56 Global cognition: n.r CMDI (total score): n.r	MMS	Self-report Informant report^d^	- 21.2% of the MS inefficient money management participants, and 9.1% of the MS efficient money management participants reported to have problems with “operating an ATM” as compared to 0% of the NC group *-* 36.4% of MS inefficient money management participants, and 31.8% of the MS efficient money management participants reported to “not often check their change” as compared to 42.3% of the NC group - 33.3% of the MS inefficient money management participants reported to “pay bills or rent late” as compared to 0% of the MS efficient money management and NC groups - 9.1% of the MS inefficient money management participants reported on “being thrown out of their accommodation” as compared to 0% of the MS efficient money management and NC groups - 42.4% of the MS inefficient money management participants reported to “owe money for debts” as compared to 0% of the MS efficient money management and NC groups - 39.4% of the MS inefficient money management participants, and 4.5% of the MS efficient money management participants reported to “spend all their money in the first few days” as compared to 3.8% of the NC group - 24.2% of the MS inefficient money management participants reported to “go without essentials because of having no money left” as compared to 0% of the MS efficient money management and NC groups - 30.3% of the MS inefficient money management participants, and 4.5% of the MS efficient money management participants reported to “engage in problematic impulse buying” and to “spend all their money on things they like” as compared to 0% of the NC group - 57.6% of the MS inefficient money management participants reported to “need to borrow money” as compared to 0% of the MS efficient money management and NC groups	The percentage of participants reporting problems in money management was highest in the MS inefficient money management group for all items of the MMS, except for the item “don’t often check change,” where the percentage of NC participants reporting problems was highest For all items of the MMS, the percentage of participants reporting problems in money management in the MS efficient money management group was (highly) similar to the percentages observed in the NC group
		**MS inefficient money management** (*n* = 38) Age (y): 50.1 ± 7.9 Sex (m/f): 8/30 Education (y): 15.5 ± 1.9	MS subtypes: - RR (80%) - PP (17.5%) - SP (2.5%) Disease duration (mo): 198.3 ± 121.9 MSFC (*z*-score): − 0.36 ± 0.68 Global cognition: n.r				
		NC (*n* = 26) Age (y): 44.4 ± 10.5 Sex (m/f): 9/17 Education (y): 17.2 ± 1.8	MSFC (*z*-score): 0.17 ± 0.43 Global cognition: n.r CMDI (total score): n.r				

*ATM* automated teller machine, *AR* actual reality, *CMDI* Chicago Multiscale Depression Inventory, *CS* cross-sectional design, *d* Cohen’s *d* (effect sizes were calculated and converted making use of www.psychometrica.de), *FBP* Functional Behavior Profile, *IADL* instrumental activities of daily living, *k* number of studies, *m/f* male/female ratio, *MMS* Money Management Survey, *mo* number of months, *MS* multiple sclerosis, *MSFC* Multiple Sclerosis Functional Composite;* n* sample size, *NB* nota bene, *NC* normal control (group), *n.r.* not reported, *PP* primary progressive type, *RR* relapsing remitting type, *SP* secondary progressive type, *USA* United States of America, *y* number of years

^a^All descriptive variables are reported as mean ± standard deviation, unless otherwise indicated

^b^Refers to the source (self or informant report) of the relevant patient group(s) only, unless otherwise specified

^c^ > and < refer to significant differences only, based on the alpha levels used in the original studies

^d^Scores not reported for informant report version of MMS

**Table 8:**
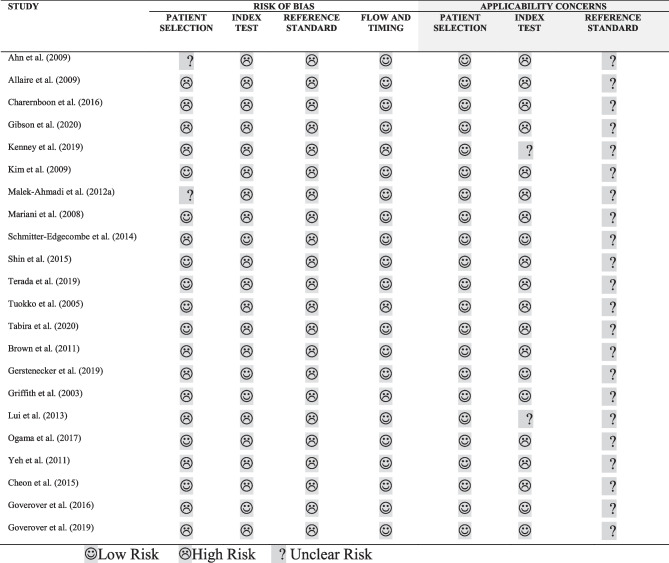
Quadas-2 quality assessment and risk of bias evaluation of included studies

When exploring the literature on financial capability and neurodegenerative diseases, it becomes clear that the terms financial ability, competency, capacity, and capability have been used almost interchangeably with slight differences in meaning across disciplines (Appelbaum et al., 2016). Within the context of the present study, terminology will be used as introduced by Appelbaum et al. (2016), in order to ensure clarity and consistency throughout the text. Appelbaum et al. (2016) defined financial capability as the management of one’s finances in a way that serves one’s personal needs and goals. In the evaluation of financial capability, a distinction can be made between financial competence and financial performance. According to the definition of Appelbaum et al. (2016), financial competence is generally assessed in a controlled setting and refers to an individual’s financial skills as reflected by their financial knowledge (e.g., their knowledge about the concept of money or online banking procedures) and their financial judgment. Financial performance, on the other hand, reflects real-world functioning and refers to an individual’s degree of success in dealing with financial demands, issues, or questions in the context of all stressors and resources in their personal environment. Successful financial performance thus requires both sufficient financial competence, as well as the presence of the abilities to implement financial decisions, and the possibility to use these abilities in everyday life (Appelbaum et al., 2016).

A recent systematic review and meta-analysis by Bangma et al. (2021) showed that people living with neurodegenerative diseases consistently present with more problems on performance-based financial tasks than healthy individuals, and that the degree of these problems seems to be related to the severity of cognitive decline. Indeed, minor cognitive difficulties that accompany normal aging might already have a negative influence on financial task performance and financial literacy (Bangma et al., 2017; Finke et al., 2017), although normal aging could also positively influence performance on financial tasks due to increased financial knowledge and experience, and the more stable affective processing that is associated with advancing age (Bangma et al., 2017). However, while such performance-based financial tasks may form an adequate measure of financial competence, relatively little can be said still, based on these studies, regarding the everyday financial performance of people living with neurodegenerative diseases. As the contextual factors in an individual’s environment can have a positive as well as a negative influence on their financial performance, a discrepancy may exist between an individual’s performance on structured financial tasks as applied in a controlled setting and their financial performance in daily life (Appelbaum et al., 2016). For example, if an individual with limited financial competence is supported by others in dealing with their financial matters (e.g., a caregiver sets up automatic bill-paying for rent and other necessities), they may show deficiencies on financial competence tasks, but still be successful in their everyday financial performance. If, on the other hand, an individual for example suffers from depression, this does not necessarily affect their financial competence either, but the depressive symptoms could negatively impact their ability to meet the financial demands of daily life (Appelbaum et al., 2016). Because of this potential discrepancy between task and daily-life performance, self and informant reports of an individual’s degree of success in dealing with their everyday financial demands, issues, or questions form an important, complementary source of information to performance-based financial competence tasks in determining financial capability. Moreover, self and informant reports can be used to assess financial capability at a different level than performance-based financial tasks. While performance-based tasks usually take place in a highly controlled setting, where individuals are asked to maximize their performance, self and informant report measures often require the individual to rate their daily-life performance over, for example, the past month (Fuermaier et al., 2015). Thus, whereas performance-based measures reflect an individual’s optimal performance at the moment of assessment, self and informant report measures provide information about an individual’s typical performance, averaged over longer periods of time (Fuermaier et al., 2015; Toplak et al., 2013). Indeed, previous research shows that subjective (self or informant report) and objective (performance-based) measures do not necessarily assess the same constructs (Fuermaier et al., 2015; Koerts et al., 2012; Toplak et al., 2013), suggesting that the use of self and informant report measures can offer distinct and valuable information about the financial capability of people living with neurodegenerative disease.

In this context, it is important to mention, however, that as compared to performance-based tasks, self and informant report measures rely more strongly on adequate insight into the daily-life functioning of an individual (Wadley et al., 2003). In people living with neurodegenerative disease, cognitive decline can cause reduced insight into one’s financial abilities (Gerstenecker et al., 2019), which may consequently lead to over or underestimations of their own financial performance. In a like manner, informant reported measures are susceptible to inaccuracy as informants may not always have the opportunity or time to make reliable observations of an individual’s current financial performance (Appelbaum et al., 2016). Given their susceptibility to bias, it should thus be emphasized that self or informant reports must be used to complement rather than substitute performance-based financial tasks. As shown in Table 1, measures of financial competence and financial performance have different strengths and limitations, and information on both types of measures is needed to gain insight into the financial capability of people living with neurodegenerative disease.

As the aforementioned study by Bangma et al. (2021) has reviewed studies that evaluated the performance on financial competence tasks of people living with neurodegenerative diseases, the aim of this study thus is to complement their findings by providing an overview of the literature examining self and informant report assessments of financial performance in these patient groups. By addressing assessments of financial performance, this study can provide valuable insight into the everyday financial functioning of people living with neurodegenerative disease in the context of their personal environment (see Table 1). In combination with the knowledge we have about financial competence assessments (Bangma et al., 2021), this will add to our understanding of the potential strengths and weaknesses in financial capability of people living with neurodegenerative disease, which is needed to develop and offer tailored support. The specific aim of this study thence was to determine the type and extent of subjectively reported problems in financial performance of people living with different neurodegenerative diseases as compared to the reports of a (cognitively) normal control group or as compared to an earlier point in time. If possible, the self and informant reports of financial performance were also compared between people living with different neurodegenerative diseases. A further aim was to explore what measures or variables are associated with the financial performance of people living with neurodegenerative disease. To this end, the existing literature on neurodegenerative diseases and self or informant reported financial performance has been systematically searched and analyzed.

## Method

### Study Selection Procedure

A systematic search of the available literature addressing financial capability (including financial performance) and neurodegenerative diseases was carried out according to the guidelines of the Preferred Reporting Items for Systematic Reviews and Meta-Analyses (PRISMA; Moher et al., 2009). Journal articles were searched through the databases PsycINFO, MEDLINE, PubMed, and Web of Science. Primary keywords for the literature search were related to neurodegenerative diseases, and included, for example, Parkinson’s disease, Alzheimer’s disease, mild cognitive impairment, *OR* dementia. Secondary keywords were related to financial capability, and included terms such as financial performance, finances, *OR* money management (for a complete list of primary and secondary keywords and further clarification of the chosen keywords; see Appendix 1). A combination of primary and secondary keywords (e.g., dementia [*AND*] financial performance) had to appear in either the title or abstract of the articles. Only peer-reviewed articles that were written in English were included in the review.

The selection criteria for this systematic review were as follows: studies were included when they (a) included at least one relevant patient group that was diagnosed according to published criteria (e.g., according to DSM-5 or ICD-10 criteria), (b) included a group comparison between the relevant patient group(s) and a (cognitively) normal control group ((C)NC group) or adopted a longitudinal design, and (c) reported the outcomes of a structured self or informant report assessment of financial performance (e.g., a questionnaire or interview) or of a structured self or informant report measure that includes a subscale of at least one item on financial performance. Studies were excluded when they (a) only included a mixed patient group (e.g., a “dementia” group), (b) primarily focused on financial risk taking (e.g., impulsive buying), (c) primarily focused on (pharmacological) intervention effects, or (d) primarily focused on self or disease-awareness of the patient group(s) (i.e., reported solely on the relation or discrepancy between objective and subjective measures or between self-reported and informant reported measurements). Self or informant reported assessments were considered measures of financial performance only if they directly addressed an individual’s degree of success in dealing with their everyday financial demands, issues, or questions (after Appelbaum et al., 2016). Measures that were indirectly related to financial performance, such as questions on financial status or whether an individual has a representative payee, were not considered in this review. Additionally, items on (independence in) shopping were also not included as the abilities required to shop independently certainly include, but are not limited to, financial skills only. For example, in the more detailed Instrumental Activities of Daily Living – Compensation scale (IADL-C) (Schmitter-Edgecombe et al., 2014), one of the items that refers to shopping skills includes whether the individual is able to efficiently plan the sequence of stops on a shopping trip.

**Fig. 1:**
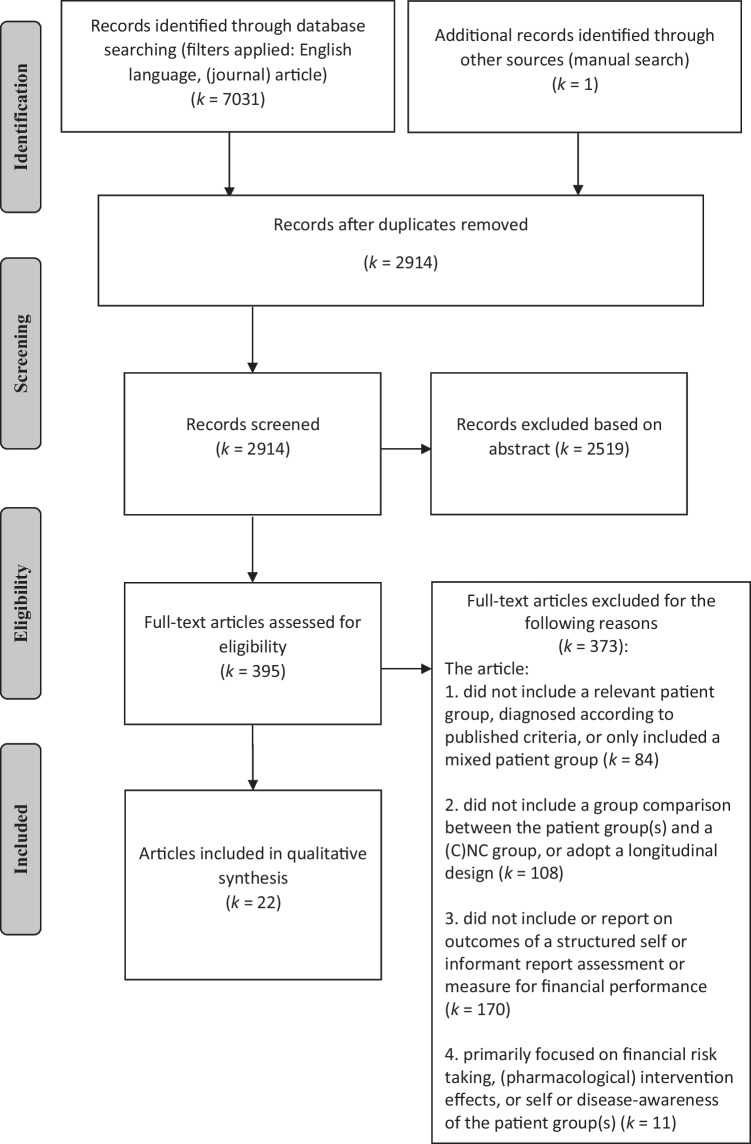
Flow diagram of the systematic search and review process according to the guidelines of Preferred Reporting Items for Systematic Reviews and Meta-Analyses (PRISMA; Moher et al., 2009)

The literature search for the systematic review was finalized on the 12th of February 2021, resulting in a literature list of 7031 articles, from which duplicates were removed (see Fig. 1). The retrieved literature was supplemented with relevant literature cited in the articles found (manual search; Fig. 1). Titles and abstracts of a remaining 2914 studies were screened to see whether these studies addressed the topic at hand. After this screening, 395 studies remained, which were read in full in order to identify those articles that did not fulfill the selection criteria listed above. These articles were subsequently excluded (see Fig. 1). In total, 22 studies were included in the review.

### Content Analysis

After the study selection procedure was completed (see Fig. 1), a content analysis was conducted for the included studies. The results were extracted and organized in table format according to diagnosis of the relevant patient group(s), including the following aspects; first author, country and year of publication, study design, sample characteristics (i.e., demographic and disease characteristics), adopted financial performance assessment, measure or item, source (i.e., self or informant report), and the main study outcomes considered relevant for the research question at hand. Relevant study outcomes included outcomes of comparisons between neurodegenerative disease group and a (C)NC group or comparisons between different neurodegenerative disease groups and longitudinal analyses regarding the financial performance assessments, as well as reported associations of other measures/variables with the financial performance assessments used. If a study included more than one participant group, only those groups for which outcomes on the financial performance assessment were reported and were described in Tables 3, 4, 5, 6 and 7. In line with the inclusion and exclusion criteria, (comparisons with) mixed patient groups (e.g., a dementia group) were not listed in Tables 3, 4, 5, 6 and 7, as it is not possible to draw clear conclusions about individuals with a specific neurodegenerative disease based on this information. Study outcomes were all reported in close accordance with the results reported in the original studies, and, if reported on, group differences were considered significant at the alpha levels used in the original studies. If possible, effect sizes were calculated for the relevant group comparisons based on reported statistics or group means, making use of www.psychometrica.de (Lenhard & Lenhard, 2016). Via this website, all effect sizes were converted to Cohen’s *d*. Effect sizes in the order of 0.2, 0.5, and 0.8 can be interpreted as small, medium, and large, respectively (Cohen, 1969).

### Quality Assessment and Risk of Bias

**Fig. 2:**
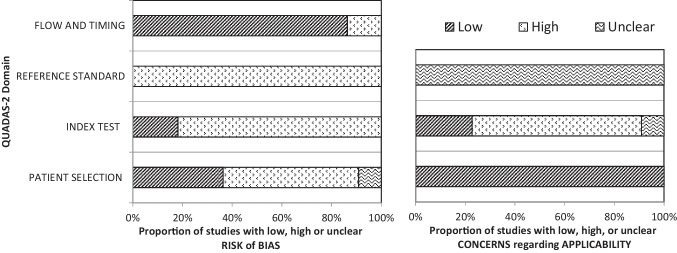
Graphical display of results Quadas-2 quality assessment and risk of bias evaluation

A modified version of the QUADAS-2 tool (Whiting et al., 2011) was used to assess the methodological quality and risk of bias of each of the included studies. The QUADAS-2 tool can be used to assess risk of bias in four key domains: (1) patient/participant selection, i.e., the selection of the neurodegenerative disease and (C)NC groups; (2) index test, i.e., the financial performance assessment; (3) reference standard, i.e., the use of normative data or pre-defined cut-offs for the level of financial performance; and (4) flow and timing, i.e., the flow of the participants through the study, assessments, and analyses. The QUADAS-2 was originally designed to assess the quality of diagnostic accuracy studies (Whiting et al., 2011), but, as recommended by the authors, the signaling questions used to help reach judgment on the risk of bias were tailored to better fit with the type of studies included in the present review. The signaling questions used to assess the risk of bias for each of the QUADAS-2 domains are provided in Appendix 3 (Table 11). Based on the answers to these signaling questions and narrative descriptions of the results, the risk of bias within the included studies was judged as being high, low, or unclear for each of the four domains. The first three QUADAS-2 domains (i.e., patient selection, index test, and reference standard) were furthermore considered in terms of the level of concern regarding applicability to the review question. The level of concern regarding applicability was also rated as high, low, or unclear. We did not aim to exclude studies based on the risk of bias or applicability judgments. Further, as recommended by the authors, the QUADAS-2 tool was not used to generate a numerical quality score (Whiting et al., 2011). Instead, the results of the QUADAS-2 assessments were summarized in table format for each of the individual studies (see Table 8), and overall results were displayed graphically in Fig. 2.

## Results

In total, 22 studies were included in this review (see Fig. 1; Appendix 2 (Table 10)). All studies made use of a self or informant reported assessment, measure, or subscale of at least one item to study financial performance in people living with MCI (*k* = 18), AD (*k* = 8), PD (*k* = 1), or MS (*k* = 2). Regarding the use of questionnaires or subscales on financial performance, 14 studies (i.e., 64%) made use of a subscale that included 1 to 3 items on financial performance, one study (i.e., 5%) made use of a subscale of more than three financial performance items, and four studies (i.e., 18%) used a full questionnaire, addressing various aspects of financial performance. Three studies (i.e., 14%) used a different form of assessment for financial performance, i.e., clinician ratings of the “level of financial independence” (Kenney et al., 2019) and the “mental competence to make financial decisions” (Lui et al., 2013), and family reports of the occurrence of specific problems with financial management (Terada et al., 2019). Seven of the included studies (i.e., 32%) only described the outcomes of a self-report assessment, 13 studies (i.e., 59%) only described the outcomes of informant reported assessments for the patient group(s), and one study (i.e., 5%) described both self and informant reported outcomes on the financial performance assessment. For one study (i.e., 5%), it was unclear, based on the information provided, whether the financial performance assessment was self or informant reported.

A detailed description of all instruments, subscales, or items on financial performance used in the included studies is provided in Table 2. An overview the study characteristics and relevant study outcomes is given in Tables 3, 4, 5, 6 and 7. The overall interpretation of the most important study outcomes for each of the individual studies is summarized in the Conclusion column of Tables 3, 4, 5, 6 and 7, and a synthesis of the outcomes in the context of the present review is laid out in-text. Finally, the results of the QUADAS-2 quality assessment and risk of bias evaluation are described in-text, summarized in Table 8, and displayed graphically in Fig. 2.

### Financial Performance in People Living with MCI

#### Characteristics of Included MCI Groups

Eighteen studies investigated self or informant reported financial performance of people living with MCI (see Tables 3 and 5), evaluating a total of 2382 MCI participants. The mean age of the participants ranged from 67.1 to 82.9 years (weighted average = 70.7 years). Since Kenney et al. (2019) only reported on the descriptive characteristics of their total sample, it must be noted that the mean age of the MCI group in their study could not be included in the calculation of the weighted average.

For all 18 studies, the presumed MCI etiologies of the participant groups were either unknown or not reported. The diagnostic criteria for MCI that were applied in the different studies generally distinguished between two major clinical subtypes of MCI: amnestic MCI (aMCI) and non-amnestic MCI (naMCI). Depending on the number of cognitive domains that are impaired, these clinical subtypes can be further divided into single-domain aMCI or naMCI, or multiple-domain aMCI or naMCI (Petersen, 2004; Winblad et al., 2004). Eight studies (i.e., 44%) included people living with single or multiple-domain aMCI, and one study (i.e., 6%) included both an aMCI and an naMCI group. In four studies (i.e., 22%), the MCI group was mixed (including aMCI and naMCI participants), and five studies (i.e., 28%) did not report on the clinical subtypes of MCI included in the MCI group(s).

#### MCI Compared to Cognitively Normal Controls

Comparing self or informant reported financial performance of people living with MCI and cognitively normal controls participants (CNCs), the results of 15 of the 18 studies (i.e., 83%) indicated that more problems in financial performance were reported for/by people living with MCI than for/by cognitively normal controls. Thirteen studies found significant differences between the reported financial performance for the MCI and cognitively normal control groups, and two studies did not report on significance levels between the cognitively normal control and MCI groups, but results were in line with the MCI group having more problems in financial performance than the cognitively normal controls (Brown et al., 2011; Kenney et al., 2019). Effect sizes (Cohen’s *d*) for the comparisons between the MCI and cognitively normal control groups could be derived for eight of these 15 studies and ranged from small to large, with the majority being considered as medium effects (see Tables 3 and 5). The type of problems in financial performance reported for/by people living with MCI were diverse and included problems with paying bills and handling money (e.g., calculating change), the organization of financial or tax records, finance and correspondence, and financial management (e.g., writing checks, balancing a check book, managing a budget or business affairs). In line with this, problems were reported for people living with MCI in “global financial capacity” and on several domains of the current financial capacity form (CFCF) (Gerstenecker et al., 2019; Griffith et al., 2003). Several studies furthermore reported people living with MCI to need (more) assistance in or to be (more) dependent on others in their financial management (see Tables 3 and 5). Finally, in three of the 18 studies (i.e., 17%) comparing the reported financial performance of MCI participants and cognitively normal controls, no (significant) between-group differences were found. Effect sizes (Cohen’s *d*) in these studies (*k* = 3) could be interpreted as negligible or small (Ahn et al., 2009; Lui et al., 2013; Ogama et al., 2017).

Looking at the self and informant reported assessments separately, all six studies (i.e., 100%) that described the outcomes of a self-report assessment found that significantly more problems in financial performance were reported by people living with MCI than by cognitively normal controls. Of the 12 studies that described the outcomes of informant reported assessments for the MCI groups, eight studies (i.e., 67%) found significant differences between the MCI and cognitively normal control groups, while two more studies (i.e., 17%) did not report on significance levels between the MCI and cognitively normal control groups, but the results were in line with the MCI group having more problems in financial performance than the cognitively normal controls (Brown et al., 2011; Kenney et al., 2019). Finally, two studies (i.e., 17%) did not find significant between-group differences in the informant reports. For the study by Ogama et al. (2017), it was unclear, based on the information provided, whether the financial performance assessment was self or informant reported, but this study yielded no significant differences between the MCI and cognitively normal control groups.

#### Longitudinal Findings

Apart from a cross-sectional comparison between the MCI and cognitively normal control groups at baseline, Tuokko et al. (2005) also applied a longitudinal design, by comparing the MCI group to a cognitively normal control group at two points in time, with a five-year interval. Although sample sizes were very small for the longitudinal analysis, and results should be viewed with caution, they concluded that, among the people not reporting impairments in their money management at baseline, a significantly larger percentage of people living with MCI than cognitively normal controls reported impairments in this area after five years (see Table 3).

### Financial Performance in People Living with AD

#### Characteristics of Included AD Groups

Eight studies investigated self or informant reported financial performance of people living with AD (Tables 4, 5 and 6), evaluating 824 AD participants in total, with a mean age range of 71.0 to 82.2 (weighted average = 77.4 years). Six of these studies (i.e., 75%) only included people living with (very) mild AD. In one study (i.e., 13%), participants in various stages of AD were included and subclassified for later analyses according to total MMSE scores (MMSE range: 15–30), while in another study (i.e., 13%), the disease stage of the AD group was not specified.

#### AD Compared to Cognitively Normal Controls

All eight studies (i.e., 100%) found the reported financial performance to be worse for people living with AD than for cognitively normal controls. The effect sizes (Cohen’s *d*) for the comparisons between the AD and cognitively normal control groups could be derived for six of the eight studies and ranged from small to large, with the majority of studies finding large effects (see Tables 4, 5 and 6). Of the six studies (i.e., 75%) that only included people living with (very) mild AD, five studies found significant group differences, and one study did not report on significance levels between the AD and cognitively normal control groups, but results were still in line with the mild AD group having more problems in financial performance than the cognitively normal controls (Brown et al., 2011). Reported problems in financial performance for the (very) mild AD groups included problems in handling finances, the organization of financial records, financial management (e.g., writing checks, paying bills, managing check book), tax management, and all domains of the CFCF (see Table 5). As compared to cognitively normal controls, more individuals living with mild AD were furthermore found to need assistance in or to be incapable of financial management (Griffith et al., 2003) or rated by clinicians to be mentally incompetent to make daily financial decisions (Lui et al., 2013). Including a group of people living with AD for whom the disease stage was not specified, Cheon et al. (2015) found that the scores on the managing finances item were significantly higher (indicating poorer functioning) for the AD group than for the NC group. Lastly, Ogama et al. (2017) included people in various disease stages of AD and also concluded that significantly more problems in financial performance were reported for people living with AD as compared to cognitively normal controls. Subclassifying their sample into three groups according to total MMSE score furthermore led the authors to conclude that the ability to handle finances decreases significantly with increasing cognitive impairment (see Table 5). Looking at the source types that were used, seven of the eight studies that investigated the financial performance of people living with AD described the outcomes of an informant reported assessment for financial performance (Tables 4, 5, 6 and 7), one study additionally described the outcomes of a self-report assessment (Gerstenecker et al., 2019), and for one study it was unclear, based on the information provided, whether the assessment was self or informant reported (Ogama et al., 2017). Irrespective of the source type used, all eight studies (i.e., 100%) found the reported financial performance to be worse for people living with AD than for cognitively normal controls.

#### (Mild) AD Compared to MCI

Six studies investigated differences in financial performance between individuals diagnosed with aMCI and people living with AD (see Table 5). The results of all six studies (i.e., 100%) indicated that more problems in financial performance were reported for/by the (mild) AD groups than for/by the aMCI groups.

### Financial Performance in People Living with PD

The study by Cheon et al. (2015) investigated informant reported financial performance in 72 people living with PD, including a group of cognitively normal participants living with PD and a participant group living with PDD (see Table 6). The results showed that the reported financial management abilities did not differ significantly between the cognitively normal PD group and an NC group (negligible to small effects). However, the reported difficulty was found to be significantly higher in the PDD group than in the PD and NC groups (medium to large effects). Furthermore, whereas the reported financial performance of the PDD group did not differ significantly from an AD group regarding the actual scores (small effect), the cognitive scores (i.e., scores corrected for having a motor disability) of the PDD group were significantly lower (indicating better functioning) than those of the AD group (large effect).

### Financial Performance in People Living with MS

Two studies were included that looked at the difference in self-reported difficulties in financial performance between people living with MS and NC participants (NCs) (see Table 7). In total, 102 people living with MS participated in these studies, and the mean age of the participants ranged from 47.9 to 51.6 (weighed average = 50.0 years). Goverover et al. (2016) concluded that people living with MS reported significantly more problems in money management than NCs, as reflected by the total scores on the Money Management Survey (MMS) (medium effect). On item level, the MS group showed a significantly greater likelihood than the NCs to have increased problems with regard to operating an ATM, owning debt for bills they have not paid, and needing to borrow money. Goverover et al. (2019) split their sample of MS participants into two groups, including an inefficient and an efficient money management MS group. As the cut-off for group assignment was partly based on the scores on the MMS itself, it was not surprising that the percentage of participants reporting problems on the MMS was higher in the MS inefficient money management group as compared to the MS efficient money management group and the NCs on all but one item (i.e., “don’t often check change”). In contrast, the percentages of participants in the MS efficient money management group reporting problems on the MMS items were low, and highly similar to the percentages found in the NC group. According to the authors, these results suggest that not all individuals living with MS have problems with money management, and that the money management abilities of some people living with MS may be comparable to those of individuals without MS.

### Associations of Financial Performance with Demographics, Disease Characteristics, Neuropsychological Measures, and Other Variables

#### Associations with Financial Performance in People Living with MCI or AD

##### Gender

Two studies found that in the MCI group, (possibly) combined with the cognitively normal control group, women reported significantly more problems in financial performance than men (Kim et al., 2009; Tuokko et al., 2005; see Table 3). Contrastingly, Lui et al. (2013) found that, when combining the cognitively normal control, aMCI, and mild AD groups from their study, no significant difference in gender ratio was observed between individuals who were found to be mentally competent to make daily financial management decisions, and those who were found to be mentally incompetent.

##### Age

Tabira et al. (2020) found that for both cognitively normal elderly and people living with very mild AD, the independence in the ability to handle finances decreased with advancing age. Importantly, this decrease in independence was found to start in a younger age group in people living with AD than in cognitively normal controls, and the decreasing slope with age was steeper in the very mild AD group than in the cognitively normal control group. In contrast, two other studies performed regression analyses (see Tables 3 and 5 for an overview of included variables) in a combined sample of MCI participants and cognitively normal controls (Tuokko et al., 2005), or a combined sample of aMCI and AD participants (Ogama et al., 2017), but did not identify age as a significant independent risk factor/predictor for reported problems on the financial performance item.

##### Cognitive Functioning

Charernboon and Lerthattasilp (2016) examined the relationship between MMSE scores and financial performance in participants without cognitive impairments, with MCI, and with mild, moderate, or severe dementia (diagnoses not specified). When looking for the optimum cut-off score, the authors found that a score of 24 or lower on the MMSE had the best sensitivity (0.84) and specificity (0.97) for differentiating individuals who were able versus unable to independently organize their finances. The organization of finances was therewith shown to be the first activity of daily living to be impaired along the stages of cognitive decline. Combining the aMCI and AD groups, Ogama et al. (2017) performed a multiple logistic regression analysis to identify independent risk factors for impairments in the ability to handle finances. In their first model, they found white matter hyperintensity in the frontal lobe to the only significant predictor (see Table 5 for an overview of included variables). In their second model, several variables were added as potential risk factors, rendering white matter hyperintensity in the frontal lobe as an insignificant predictor, but MMSE scores as a significant predictor of financial performance. This significant association between MMSE scores and the financial performance item seems to be in line with the finding that more problems were reported in financial performance for AD participants with moderate and lower MMSE scores (20–23 and 15–19) than for AD participants with relatively high MMSE scores (24–30), people living with aMCI and cognitively normal controls (Ogama et al., 2017). Contrastingly, two other studies did not find a significant association between MMSE scores and financial performance in the aMCI, mild AD (Gerstenecker et al., 2019), or cognitively normal control groups (Gerstenecker et al., 2019; Mariani et al., 2008).

Finally, the separate analyses performed by Mariani et al. (2008) in the aMCI and cognitively normal control groups revealed no significant associations between financial performance and comorbid illnesses, or performance on neuropsychological tests for episodic memory, language, attention/executive functioning and praxis. In contrast, Tuokko et al. (2005) found that, in a combined group of cognitively normal control and MCI participants, seven neuropsychological tests in four cognitive domains (i.e., memory, verbal abilities, visuo-constructional ability, and attention and processing speed) taken together in one model could significantly predict present financial performance. Performance on these same seven tests at T1 furthermore contributed significantly to the prediction of future difficulty in handling finances at T2 (i.e., five years later), and specifically, individuals with poorer memory at T1 were shown to be more likely to have difficulty in financial performance at T2.

#### Associations with Financial Performance in People Living with MS

Goverover et al. (2016) found that in the MS group (possibly combined with the NC group), more problems in financial performance were significantly associated with a higher reported dysfunction on an instrumental activities of daily living (IADL) scale, and on a questionnaire for everyday life task performance, social interactions, and problem-solving. Self-reported financial performance scores were not significantly associated, however, with affect symptomatology, or with performance-based test scores in the domains of learning and memory, executive functions, and processing speed and working memory.

### Quality Assessment and Risk of Bias

The methodological quality and risk of bias of all 22 included studies was assessed using a modified version of the QUADAS-2 tool (Whiting et al., 2011; see Appendix 3 (Table 11)). The results of the QUADAS-2 assessments for each of the individual studies are summarized in Table 8. Regarding the patient/participant selection, twelve studies (i.e., 55%) were judged as having a high risk of bias, eight studies (i.e., 36%) as having a low risk of bias, and for two studies (i.e., 9%) the risk of bias was unclear. The mixed results in this domain were mostly due to differences in the recruitment method for the neurodegenerative disease group(s). Most of the studies with a high risk of bias did not use a random or consecutive sampling method for the participant groups, while most studies with a low risk of bias did include a consecutive patient sample. Using a consecutive sampling method can reduce the risk of bias in a study as all eligible participants at the same hospital or clinic, for example, will have an equal chance of being included in the study, regardless of their performance on the index test. Contrastingly, the studies with a high risk of bias regarding the patient/participant selection mostly made use of purposive or convenience sampling. While widely adopted in patient studies, convenience sampling methods are prone to various forms of research bias and provide with a less reliable representation of the population than random or consecutive samples. For example, eligible patients who present with more cognitive impairments, or potentially more problems on the index test, might be less eager to volunteer for participation and could therefore be less likely to be represented in the recruited sample.

With regard to the index test (i.e., the financial performance assessment), the risk of bias was judged as being high for eighteen studies (i.e., 82%) and as low for four studies (i.e., 18%). For the majority of included studies, the risk of bias was high because the index test was considered inappropriate (i.e., too limited) to assess the multidimensional construct of financial performance. Furthermore, none of the included studies clearly described the use of a reference standard, such as normative data or a pre-defined cut-off for the level of difficulty in financial performance of the participant groups. The risk of bias with regard to the reference standard was therefore judged as being high for all included studies (i.e., 100%). In terms of flow and timing, nineteen studies (i.e., 86%) were judged as having a low risk of bias, as (almost) all participants in these studies both received the index test (i.e., the financial performance assessment) and were included in the analyses for the index test. For three of the included studies (i.e., 14%), the flow of the participants through the study, including the assessments and analyses, leads to a high risk of bias. Regarding the judgments of applicability, there was a low level of concern for all included studies (i.e., 100%) that the included patients did not match the review question. This was due to the fact that the selection criteria of the present review stated that only patient groups diagnosed according to published criteria were included, and that mixed patient groups (e.g., dementia groups) were excluded. Contrastingly, there was a high level of concern for the majority of included studies (i.e., 68%) that the index test differed from the review question at hand. While this review aims to address the construct of financial performance, the majority of index tests used were judged as being too limited to assess this broad construct in full. Finally, as no clear reference standard was defined or described in any of the included studies, the applicability to the review question of the reference standard was considered to be unclear for all 22 studies (i.e., 100%). The overall results of the risk of bias evaluation, including the judgments of applicability, are displayed graphically in Fig. 2.

## Discussion

In the evaluation of financial capability, a distinction can be made between financial competence and financial performance. While financial competence is usually assessed in a controlled setting by means of performance-based financial tasks, financial performance reflects real-world functioning, and refers to an individual’s degree of success in dealing with the financial demands, issues, or questions of everyday life (Appelbaum et al., 2016) (see also Table 1). As a previous study by Bangma et al. (2021) reviewed studies evaluating the performance on objective financial competence tasks of people living with neurodegenerative diseases, the aim of the present review was to complement their findings by providing a systematic overview of the literature examining self and informant reported financial performance in these patient groups. In total, 22 studies were included that all compared the reported financial performance of people living with an neurodegenerative disease to a (C)NC group. The vast majority of the studies included people living with MCI of unknown etiologies or people living with AD (*k* = 19), one study focused on PD(D), and two studies addressed financial performance in people living with MS. Apart from between-group comparisons, associations between the assessments of financial performance and demographic and disease characteristics as well as neuropsychological measures and other variables were explored. Further points for discussion include a critical evaluation of the subjective assessment methods for financial performance used in the included studies, outcomes of the quality assessment and risk of bias evaluation, the limitations of the present review, and, finally, recommendations for future directions of research.

### Financial Performance in People Living with Neurodegenerative Diseases

The majority of included studies suggest that subjectively reported financial performance is poorer for people living with neurodegenerative diseases than for cognitively normal individuals. In line with the findings on objective financial competence tasks as reviewed by Bangma et al. (2021), the studies on MCI or AD furthermore indicate that the degree of self or informant reported problems in financial performance seems to be related to the severity of cognitive decline.

Representing a (transitional) state of cognitive functioning between the changes associated with normal aging and dementia, MCI is often considered a prodromal stage of AD related dementia or other types of dementia. For all included studies, the presumed MCI etiologies were either unknown or not reported. MCI is characterized by concern about a change in cognitive function, and an objective decline of cognition in one or more cognitive domain, while independence in functional activities is preserved (Albert et al., 2011; Grundman et al., 2004; Petersen, 2004; Petersen et al., 1999, 2001). Regarding this latter diagnostic criterion of MCI in particular, the Diagnostic and Statistical Manual for mental disorders (5th ed.; DSM-5) states that “the cognitive deficits do not interfere with capacity for independence in everyday activities” and that “complex instrumental activities such as paying bills or managing medications are preserved” (American Psychiatric Association, 2013, Mild Neurocognitive Disorder, para. 1). The finding of the present review, namely, that the majority of studies including people living with MCI (i.e., 83%) found the reported difficulty in financial performance to be (significantly) higher in the MCI than in the cognitively normal control groups, which was substantiated by mostly medium effect sizes, appears to be in stark contrast to this diagnostic criterion. In this context, it should also be noted, however, that the DSM-5 additionally states that for complex instrumental activities “greater effort, compensatory strategies or accommodation may be required” (American Psychiatric Association, 2013, Mild Neurocognitive Disorder, para. 1) for cognitive impairments to be considered mild, which could still be in line with the findings of this review. Indeed, the reporting of increased problems in financial performance compared to a normal control group does not necessarily correspond to a loss of independence but could also amount to the MCI groups having greater difficulty in performing daily financial tasks such as paying bills, yet still managing to do so on their own. However, several of the included studies specifically reported people living with MCI to be (more) dependent on others in their financial performance as compared to cognitively normal controls (see Tables 3 and 5) which is, in turn, clearly not in keeping with a preservation of independence in IADL as stated in the DSM-5. Constituting one of the complex IADL, financial performance therefore seems vulnerable to relatively mild impairments in cognition. Importantly, the studies using a questionnaire addressing several domains of financial performance showed that while some aspects of financial performance may indeed already be impaired in the milder stages of cognitive decline, other aspects, such as basic money skills and the comprehension of financial concepts, were generally reported to remain intact in people living with (a)MCI (Gerstenecker et al., 2019; Griffith et al., 2003). These domains thus appear to be less sensitive to mild impairments in cognition. Consistent with previous findings on objective financial competence tasks (see Bangma et al., 2021), the reported problems in financial performance for people living with MCI therefore appear to be less severe compared to the later stages of cognitive decline.

Representing the more severe stages of cognitive decline, all studies including people living with (mild) AD showed the informant reported financial performance of the AD groups to be worse than for cognitively normal controls, which was confirmed by mainly large effect sizes. As supported by the studies including a cognitively normal control, MCI, and AD group (Table 5), the findings of the present systematic review suggest a decreasing slope that may be observed in subjectively reported financial performance from normal cognition, to MCI, to (mild) AD. Specifically differentiating between MCI and severity stages of AD related dementia, the study by Ogama et al. (2017) for example showed financial performance to significantly decrease with increasing cognitive impairment as expressed by total MMSE scores. Addressing several domains of financial performance, the studies by Griffith et al. (2003) and Gerstenecker et al. (2019) further indicated all domains of the CFCF (i.e., basic monetary skills, financial conceptual knowledge, cash transactions, checkbook management, bank statement management, financial judgment, bill payment, knowledge of assets/estate, and investment decisions; see Table 2) to be affected in people living with mild AD as compared to cognitively normal controls, including the domains that were found to still be relatively intact in the (a)MCI groups. It is, however, important to note that this conclusion is based on cross-sectional studies. Longitudinal studies are needed to confirm whether such a decreasing slope regarding financial performance indeed exists when considering the transition from normal cognition, to MCI, to (mild) AD.

The findings of the three studies addressing financial performance in PD(D) and MS seem to substantiate the notion that subjectively reported financial performance is related to cognition. While, as compared to cognitively normal controls, similar levels of financial performance were, for example, reported for people living with PD who were considered cognitively normal, people living with PDD were reported to have significantly more difficulty in financial performance than the cognitively normal controls. The financial performance of people living with PDD was shown to be largely comparable to the financial performance of people living with AD (Cheon et al., 2015). In a like manner, more problems in financial performance were reported for people living with MS than for NCs (Goverover et al., 2016, 2019). Yet, Goverover et al. (2019) concluded that this finding does not apply to all people living with MS. The financial performance of some people living with MS may be comparable to the financial performance of people without MS, which could possibly be explained by differences in cognition between people living with MS.

### Associations of Financial Performance with Demographics, Disease Characteristics, Neuropsychological Measures, and Other Variables

Apart from looking at financial performance across diagnostic groups, several studies evaluated the potential relations between subjective reports of financial performance, demographic and disease characteristics, and various other subjective and objective neuropsychological measures within groups. Taking the large differences between these studies into account with regard to which relations were studied, which participant groups were included in the analyses, and the type of relational analyses conducted (e.g., correlational or regression analyses), no definite conclusions can be drawn based on the present review regarding these relations. Importantly, several of the included studies do however provide some indication that demographic characteristics (i.e., Kim et al., 2009; Tabira et al., 2020; Tuokko et al., 2005), global cognition (i.e., Charernboon & Lerthattasilp, 2016; Ogama et al., 2017), and specific cognitive functions (i.e., Tuokko et al., 2005) may be associated with subjective reports of financial performance in people living with different neurodegenerative diseases as well as in cognitively normal individuals. The potential influence of such variables on subjective reports of financial performance in both the present review and future studies can therefore not be ruled out. In the present review, not all included studies that observed significant between-group differences in age or gender ratio, for example, controlled for these differences in their analyses. In these studies, between-group differences in demographic characteristics may consequentially have affected relevant study outcomes, which adds to the risk of bias in terms of participant selection within these studies (see Table 8). Future research should thus aim to evaluate the characteristics, measures, and variables associated with financial performance measures in a more systematic manner in order to more firmly establish which factors may have an effect on self or informant reports of financial performance in people living with neurodegenerative disease. In this regard, future studies should also aim to address factors other than cognition or basic demographics (i.e., age or gender) that may hold relevant relations with financial performance. Factors such as prior levels of financial experience (e.g., Marson et al., 2000), financial literacy, socioeconomic status (see Appelbaum et al., 2016), and income level (Bangma et al., 2017), for example, have been associated with aspects of financial capability, yet, based on the included studies, their influence on the financial performance of people living with neurodegenerative diseases still remains to be explored.

### Evaluation of Subjective Assessments for Financial Performance

Financial performance constitutes a broad and multidimensional construct that may include numerous financial tasks such as paying bills, budgeting, or taking out insurances, thus requiring various conceptual, procedural, and judgmental financial skills as well as more general affective and cognitive abilities (Appelbaum et al., 2016). Referring specifically to an individual’s real-world performance (Appelbaum et al., 2016), it becomes clear, based on the definition alone, that the concept of financial performance cannot be fully captured in one single questionnaire item or subscale. However, of the studies included in the present review, the majority made use of 1 to 3 self or informant report items that addressed financial performance in people living with neurodegenerative diseases, specifically inquiring about specific aspects of financial performance only (e.g., difficulty/independence in paying bills; see Table 2). Consequently, these studies all pose a high risk of bias in terms of the assessment used (i.e., the index test) as the adopted methods are considered inappropriate to address the broad construct of financial performance (see Table 8; Fig. 2). This limited manner of assessing subjectively reported financial performance is particularly problematic as the outcomes of the present review indicate that people living with different neurodegenerative diseases report to/are reported to experience more difficulty in this area than cognitively normal individuals. Clearly, the current assessment methods used cannot comprise such problems in full.

Indeed, only four of the included studies made use of an entire questionnaire solely focused on financial performance, providing a more comprehensive picture of the difficulties in financial performance reported for/by people living with neurodegenerative disease (Gerstenecker et al., 2019; Goverover et al., 2016, 2019; Griffith et al., 2003). First, the MMS (Hoskin et al., 2005; see Table 2), used in the studies by Goverover et al., (2016,2019), consists of 11 factual questions about difficulties in daily money management activities, making the questionnaire relevant for the identification of concrete problems in financial performance. At the same time, the MMS appears less fitting for the evaluation of the relative strengths in financial performance an individual may have, as the items are all phrased in a rather negative manner (e.g., “do you pay the bills late?”). The questionnaire furthermore includes a few items that specifically inquire about financial situations that could be considered less common or more extreme. The item on whether someone has lost their accommodation for not paying rent in particular seems to have led to a floor effect in the study groups included by Goverover et al., (2016, 2019). A final disadvantage of the MMS as a measure for financial performance is that the questionnaire was developed using a sample of people living with acquired brain injury (Hoskin et al., ). Since this comprises a broad and heterogeneous group, the questionnaire may not necessarily be suitable for all participant groups, including people living with a specific neurodegenerative disease.

In the studies by Griffith et al. (2003) and Gerstenecker et al. (2019), the CFCF (Wadley et al., 2003) was used as a measure of financial performance. This questionnaire addresses various financial domains (i.e., basic monetary skills, financial conceptual knowledge, cash transactions, checkbook management, bank statement management, financial judgment, bill payment, and knowledge of assets/estate) on the basis of which an overall financial performance score labeled “global financial capacity” can be calculated (see Table 2). Since the CFCF includes items on complex as well as more basic financial abilities, the questionnaire seems to provide a comprehensive picture of the financial performance of an individual or a group. It should be noted, however, that some aspects of financial performance still remain unaddressed in the CFCF. Important aspects that are not considered, for example, include knowledge of personal income, regular financial liabilities, insurances, loans, or debts, the ability to set personal financial goals and plan ahead financially (e.g., planning for retirement), budgeting skills, and the ability to save money. Nevertheless, out of the assessment methods used in the studies included in the present review, the CFCF appears to be the most comprehensive instrument for financial performance that has been adopted to date. Currently, we would therefore recommend to use this instrument in research and clinical practice to assess the financial performance of people living with neurodegenerative disease. Particularly in combination with the corresponding performance-based Financial Capacity Inventory (Griffith et al., 2003; Marson et al., 2000), which addresses the same financial domains as the CFCF, both the self and informant report versions of the CFCF can provide useful insight into the strengths and weaknesses in financial capability of an individual or group. For one of the two included studies that used the CFCF (i.e., Gerstenecker et al., 2019), the effect sizes (Cohen’s *d*) for the comparisons between the three study groups could be calculated based on the information provided in the study. For the comparisons between the informant reports for the cognitively normal control, aMCI, and mild AD groups, the effect sizes for all nine financial domains and the overall financial performance score were large (see Table 5). For the comparisons between the self-reports of the cognitively normal control, aMCI and mild AD groups, the effect sizes ranged from small to large, with most effect sizes being interpreted as medium (see Table 5). Within this context, it is important to mention that the large effect sizes were predominantly determined by the comparison between the cognitively normal control and mild AD groups.

Finally, instead of using a questionnaire, item, or subscale, Kenney et al. (2019) and Lui et al. (2013) included a clinician rating. Whereas these ratings seem to have been based on comprehensive information (i.e., patient interviews or informant interviews, questionnaires and medical records), the outcome measures used to address financial performance were still limited, as they were either dichotomous (i.e., mentally competent/incompetent to make financial decisions) or trichotomous (i.e., fully dependent/needs assistance/independent in financial management). Therefore, these kinds of clinician ratings do not seem useful to fully capture the level of financial performance of people living with neurodegenerative disease.

Apart from more thoroughly addressing difficulties in financial performance, comprehensive questionnaires or interviews can also be used to shed light on the domains of financial performance that are still relatively intact in people living with neurodegenerative diseases. Such evaluations of both the relative strengths and weaknesses in financial performance of people living with neurodegenerative diseases are of the utmost importance to identify the required type and level of support, while at the same time determine domains of financial performance in which autonomy can be preserved. Whereas the present review does indicate people living with neurodegenerative diseases to be more vulnerable to impairments in financial performance than cognitively normal individuals, it should thus be emphasized that this does not necessarily apply to all domains of financial performance (e.g., Gerstenecker et al., 2019; Griffith et al., 2003), or to all (people living with) neurodegenerative diseases. In their study, Lui et al. (2013), for example, stress that despite the significance of their between-group findings, more than half of the participants living with mild AD were still found mentally competent to make day-to-day financial decisions based on the clinician ratings (see Table 5). These kinds of findings are particularly relevant in the evaluation of financial performance, as they show that having neurodegenerative disease does not mean that a person lacks the capacity to manage all aspects of their finances per se. In this context, it should also be noted that the quality assessment and risk of bias evaluation (Table 8 and Fig. 2) indicated that in addition to the use of a control group, none of the included studies clearly described the use of a reference standard, such as normative data, or pre-defined cut-offs to determine the participants’ level of financial performance. While most studies found that the neurodegenerative disease participants scored significantly worse on the financial performance assessment than the cognitively normal controls, these between-group differences do not necessarily indicate people living with neurodegenerative disease cannot perform a certain financial task, but solely that they perform worse than cognitively normal individuals. Reference standards such as normative data or cut-off scores are needed to interpret the assessment scores in the context of a condition or to determine whether scores on the financial performance assessment deviate from the financial performance of a normative sample, large enough to represent the general population. To gain further insight into the financial capability of people living with different neurodegenerative diseases, it is thence highly recommended that future studies aim to develop, adopt, and adequately report on more comprehensive assessments for financial performance and compare outcomes of these assessments to both cognitively normal controls and to normative samples that represent people living with neurodegenerative disease or the general population. Rather than using a small number of self and informant report items that inquire about specific aspects of financial performance only, comprehensive assessments could for example include questionnaires (e.g., the CFCF) or interviews that address financial performance across several domains.

### Quality Assessment and Risk of Bias

For all included studies, the methodological quality and risk of bias was assessed by making use of a modified version of the QUADAS-2 tool (Appendix 3 (Table 11)). As this tool was originally designed to assess the quality of diagnostic accuracy studies (Whiting et al., 2011), not all domains were found optimally fitting for the type of cross-sectional studies included in the present review. The domain addressing the reference standard in particular turned out not to be applicable to the included studies. As explained above, none of the included studies clearly described the use of a reference standard, which we defined as either normative data or a pre-defined cut-off for the level of financial performance. However, by tailoring the signaling questions to this review (Appendix 3 (Table 11)), the QUADAS-2 quality assessment that was carried out still provides a good indication of what aspects of the included studies pose the greatest risk of bias. Overall, the risk of bias in terms of participant selection was mixed due to the heterogeneity in recruitment methods for the neurodegenerative disease groups in particular, and the risk of bias regarding flow and timing could generally be considered low (Fig. 2). In accordance with our evaluation of the assessment methods used, as discussed above, the largest risk of bias within the included studies therefore concerned the conduct and interpretation of the index test (i.e., the financial performance assessment). For the vast majority of studies, the assessment method used was considered inappropriate (i.e., too limited) to fully address the multidimensional construct of financial performance (Fig. 2). When interpreting the results of this review, this high risk of bias in terms of the index test, as well as the absence of reference standards for the interpretation of the participants’ level of financial performance, should be taken into account.

### Limitations and Future Directions for Research

The results of the present review have to be interpreted with care as several limitations need to be acknowledged. A first limitation that manifests itself on review level is that only a limited number of studies could be included (*k* = 22) in the present review, and that the vast majority of included studies focused on people living with AD or people living with MCI. Consequently, no conclusions can be drawn, based on the present review, about the financial performance of individuals with other common neurodegenerative diseases, such as frontotemporal dementia or Huntington’s disease. This systematic review has, therefore, identified a gap in the current evidence base. More research is needed concerning subjectively reported financial performance in people living with neurodegenerative diseases in general, and neurodegenerative diseases other than AD or MCI specifically. Further, all of the included studies have adopted a cross-sectional design, while only one study additionally included a longitudinal analysis (Tuokko et al., 2005). The longitudinal analysis performed in this study can moreover be considered as suboptimal, since the sample sizes were very small, and financial performance was only studied over time in people living with MCI and cognitively normal controls who did not report impairment on the financial item at baseline (see Table 3). As the present review indicates that cognitive decline seems to be associated with decreasing financial performance, it is thus highly recommended to conduct more longitudinal research in order to enable further investigation of this association.

A second limitation concerns the high level of variability between the studies. The included studies differ greatly regarding sample characteristics, financial performance assessments, and statistical analyses used (see Tables 3, 4, 5, 6 and 7), complicating their comparability for the present review. One of the factors that may particularly contribute to the heterogeneity between studies is that the diagnostic criteria of the included neurodegenerative diseases have evolved over time. One of the more impactful changes being that the diagnostic criteria for dementia in the DSM IV-TR have been adapted to include criteria for minor and major neurocognitive disorders in the DSM-5, which was published in 2013 (American Psychiatric Association, 2013); criteria that largely correspond to the criteria for MCI and dementia, respectively. As the publication dates of the included studies range from 2003 to 2020, such adaptations in the diagnostic criteria may have influenced the results.

A third limitation on review level is that, based on the chosen keywords and inclusion criteria, not all studies have been identified that focused on IADL, or may have used an IADL item or subscale focused on financial performance. Importantly, the focus of the present review was on assessments of financial performance specifically, rather than IADL measures generally. Furthermore, many of the studies that focus on IADL do not report their outcomes on subscale or item level, but only report a total IADL score instead. In addition, as has been argued above, single items or subscales can be deemed unsuitable to address financial performance in everyday life properly and comprehensively. Moreover, part of the available IADL scales include items that focus on general shopping abilities, which, as discussed before, does not coincide fully with the definition of financial performance. Taking these considerations into account, the present systematic review can and should not be taken as a complete overview of all studies using finance-related IADL items, but rather as an overview of studies specifically addressing financial performance in people living with different neurodegenerative diseases, for which some studies made use of an IADL (sub)scale.

A fourth limitation concerns that none of the included studies formally examined the influence of factors such as ethnicity and cultural background, socioeconomic status, or prior financial experience on self and informant reported financial performance. Some of the included studies did, for example, add race, socioeconomic status, or income level as a demographic characteristic to their study, but none of the studies subsequently addressed associations between these kinds of variables and the assessment of financial performance. As factors like ethnicity, socioeconomic status, or financial experience seem to be of clear relevance to the level of financial performance of an individual, it is recommended future studies take these factors into account when examining the financial performance of people living with neurodegenerative disease.

Finally, it needs to be acknowledged that the present review has focused on self and informant reports of financial performance, which are known to be susceptible to bias (Appelbaum et al., 2016). Indeed, both the self and informant reports considered in this systematic review could potentially be over or underestimations of the actual financial performance of people living with neurodegenerative diseases. Further, discrepancies may exist between self and informant reports of financial performance, which have been suggested to be due to reduced insight of people living with neurodegenerative diseases into their financial abilities (Gerstenecker et al., 2019). It should be stressed, however, that the aim of the present review was to specifically identify what kind of problems in financial performance are reported for/by people living with neurodegenerative diseases, and to, in this way, complement rather than substitute the outcomes on performance-based financial tasks. While objective, performance-based financial tasks may form an adequate measure of financial competence and are less susceptible to bias, subjective assessments appear to be more suitable measures for the everyday financial performance of an individual or group (see Table 1). Future research should thus aim to combine both objective and subjective measures to gain further insight into the financial capability of people living with neurodegenerative diseases.

## Conclusion

This systematic literature review indicates that people living with neurodegenerative diseases are more vulnerable to impairments in financial performance than cognitively normal individuals. Furthermore, financial performance appears sensitive to relatively mild impairments in cognition. In line with the findings on the performance of people living with neurodegenerative disease on objective financial competence tasks (Bangma et al., 2021), the degree of self or informant reported problems in financial performance seems to be related to the severity of cognitive decline. As the vast majority of studies addressed financial performance in people living with MCI or people living with AD, more research is, however, needed to investigate financial performance in other neurodegenerative diseases. Further, associations between financial performance and demographic variables, global cognition, and specific cognitive functions still need to be established and should be systematically evaluated by future research. A substantial limitation of the small number of studies that report on financial performance outcomes in people living with neurodegenerative diseases (*k* = 22) is that the majority seems to assess financial performance with a single item or subscale. As financial performance constitutes a broad and multidimensional construct, these methods of assessment cannot fully comprise the problems in financial performance people living with neurodegenerative diseases might experience. Moreover, these methods do not allow for the evaluation of their relative strengths in financial performance. In order to avert adverse personal and legal consequences, identify the required type and level of support, and preserve autonomy in those domains of financial performance that are intact still, it is thus recommended that more comprehensive assessments for financial performance (e.g., detailed questionnaires or interviews) are developed and adopted. Specifically in combination with performance-based assessments of financial competence, these self or informant report assessments can offer valuable information about the strengths and weaknesses in financial capability of people living with neurodegenerative diseases.

## Appendix 1; Overview and Clarification of Chosen Keywords

**Table 9:** Overview of the primary and secondary keywords used for the literature search

**Primary search terms related to neurodegenerative diseases**	**Primary keywords (used in search)**
Neurodegenerative*	Neurodegenerative Neurodegenerative disease(s) Neurodegenerative disorder(s)
Parkinson*	Parkinson Parkinson disease Parkinson disorder Parkinson’s disease Parkinson’s disorder
Alzheimer*	Alzheimer Alzheimer disease Alzheimer disorder Alzheimer’s disease Alzheimer’s disorder
Huntington*	Huntington Huntington disease Huntington disorder Huntington’s disease Huntington’s disorder
Multiple sclerosis	Multiple sclerosis
Progressive supranuclear palsy	Progressive supranuclear palsy
Amyotrophic lateral sclerosis	Amyotrophic lateral sclerosis
*Dementia	Dementia Frontotemporal dementia Lewy bodies dementia
*Cognitive impairment	Cognitive impairment(s) Mild cognitive impairment(s)
**Secondary search terms related to financial capability**	**Secondary keywords (used in search)**
Finance	Finance Finances
Financial*	Financial Financial ability Financial inability Financial abilities Financial capability Financial incapability Financial capabilities Financial capacity Financial incapacity Financial competence Financial incompetence Financial competency Financial incompetency Financial decision(s) Financial decision(-)making Financial skills Financial judgement Financial knowledge Financial choice Financial understanding Financial reasoning Financial functioning Financial literacy Financial illiteracy Financial management Financial self-management Financial mismanagement Manage financial affairs Financial planning
Money*	Money Money management Money mismanagement

### Clarification of Chosen Keywords

#### Primary Keywords

The primary keywords of this review (i.e., all keywords related to neurodegenerative diseases; see Table 9) are broadly consistent with the primary keywords used in the aforementioned review by Bangma et al. (2021) and were selected based on the prevalence rates of the diseases. Therefore, rarer neurodegenerative diseases, such as Creutzfeldt-Jakob disease or corticobasal degeneration, were not included as keywords. Since it goes beyond the scope of the present review to elaborate on the different neurodegenerative diseases, only a short description of the neurodegenerative diseases that were considered as keywords for this review will be provided here.

With prevalence rates increasing markedly with age, AD is the leading cause of dementia and the most common neurodegenerative disease (Qiu et al., 2009). AD is characterised by an insidious onset and gradual cognitive decline that typically starts with impairments in memory and learning, but can concern various cognitive domains, inevitably leading to a progressive loss of functional independence (American Psychiatric Association, 2013).

Representing a (transitional) state of cognitive functioning between the changes associated with normal aging and dementia, MCI is often considered a prodromal stage of AD related dementia (or other types of dementia) and was, for this reason, also considered in the present review. The diagnostic criteria of MCI generally include a subjective concern about a decline in cognition, an objective impairment in one or more cognitive domain, the preservation of independence in functional activities, and cognitive impairments being sufficiently mild for the person not to be diagnosed with dementia (Albert et al., 2011; Grundman et al., 2004; Petersen, 2004; Petersen et al., 1999, 2001).

After AD, PD is the second most common neurodegenerative disease (de Lau & Breteler, 2006). Whilst the clinical diagnosis of PD is based on the associated motor-symptoms (i.e., bradykinesia, resting tremor and rigidity), non-motor symptoms, including cognitive impairments in the domains of attention and executive functioning, memory, and visuospatial functions (Kehagia et al., 2010), are often also present in people living with PD (Kouli et al., 2018). The cognitive deficits associated with PD are common even in the early disease stages (Pfeiffer et al., 2014) and can, with progression of the disease, result in PDD (Hely et al., 2005; Williams-Gray et al., 2013).

Closely related to PDD with regard to neuropathologic and clinical features, dementia with Lewy bodies (DLB) is another common neurodegenerative disease (Hogan et al., 2016). Like PD(D), DLB is characterised by non-motor symptoms, including impairments in cognition (Gomperts, 2016; McKeith et al., 2005), and motor symptoms such as bradykinesia, tremor, and rigidity. The cognitive domains that are affected in DLB largely overlap with those affected in PDD and include the domains of attention and executive functioning, memory, and visuospatial functioning (Lippa et al., 2007). An essential feature differentiating PDD and DLB is the temporal sequence of the cognitive and motor impairments. While for a diagnosis of PDD cognitive impairments develop in the context of an established PD, in DLB, the cognitive symptoms often precede—or otherwise occur within the first year after—the onset of motor symptoms (Gomperts, 2016; McKeith et al., 2005).

Another neurodegenerative disease considered as a keyword for the present review is progressive supranuclear palsy (PSP). PSP is regarded as an atypical parkinsonism and is typically characterised by ocular motor dysfunction, postural instability, akinesia (i.e., parkinsonian features) and cognitive dysfunction, particularly in the domains of attention and executive functioning (Höglinger et al., 2017; Litvan et al., 1996a, b).

Sharing some pathological and clinical features with PSP, frontotemporal dementia (FTD) is a common cause of dementia under the age of 65 (Ratnavalli et al., 2002). FTD refers to a group of disorders caused by progressive neurodegeneration of the frontal and temporal lobes of the cerebral cortex. Within the context of FTD, two main subsyndromes can be distinguished; behavioural variant FTD (bvFTD), which is the most common phenotype of FTD, and primary progressive aphasia (PPA), which can, in its turn, be divided into several subtypes (Kirshner, 2014). The hallmark symptoms of bvFTD include behavioural, emotional and personality changes, and cognitive impairments in the domains of executive functioning and social cognition (Rascovsky et al., 2011). In contrast, the subtypes of PPA are primarily characterised by impairments in language (Gorno-Tempini et al., 2011).

The neurobehavioural features associated with FTD are highly similar to those associated with amyotrophic lateral sclerosis (ALS), which was therefore also considered as a relevant neurodegenerative disease in the present review. Whereas the degeneration in ALS predominantly affects the motor system, changes in behaviour, personality or emotion, and cognitive deficits in executive functioning, social cognition, or language (Phukan et al., 2007) are also often observed in people living with ALS.

Huntington’s disease (HD) is an autosomal-dominant neurodegenerative disease that is characterised by involuntary movements, neuropsychiatric features (e.g., apathy, irritability, and depression), and cognitive decline. The cognitive deficits associated with HD include impairments in various domains such as executive functioning, social cognition, and memory (Snowden, 2017).

Finally, forming one of the major causes for neurological disability in young adults, MS was also regarded as neurodegenerative disease in the present review. Despite being generally considered as an autoimmune demyelinating disease of the central nervous system, it has been argued that the primary pathology underlying MS is a process of neurodegeneration (Chaudhuri, 2013; Stys et al., 2012). Further, the disease course of MS appears to be similar to the course of other neurodegenerative diseases. Like other neurodegenerative diseases, MS has been suggested to have a prodromal period (Wijnands et al., 2017) and often has a progressive course. Whereas it must be noted that most people living with MS will initially present with a relapsing–remitting disease course, the majority of these people will eventually develop secondary progressive MS, which is characterised by progressive atrophy of the central nervous system and neurological decline (Stys et al., 2012). MS can cause a wide range of symptoms, including fatigue, motor problems, depression, and cognitive impairments in the domains of attention and processing speed, executive functioning, and memory in particular (Jongen et al., 2012).

#### Secondary Keywords

The secondary keywords of this review (i.e., all keywords related to financial capability; see Table 9) are based on the conceptual model of financial capability as introduced by Appelbaum et al. (2016) and are broadly consistent with the secondary keywords used by Bangma et al. (2021). In order to retrieve all literature that adopted (subjective) assessments of financial performance, the list of keywords includes relevant search terms related to both finance and money.

## Appendix 2. Study characteristics

**Table 10:** Characteristics of included studies

Characteristic	*k*	Characteristic	*k*
**Total included studies**	**22**	**Study-design**	
Studies including > 1 relevant NDD	7	Between-group	21
		Longitudinal	0
		Between-group and longitudinal	1
**Year of publication**		**Number of studies per diagnosis**	
2000–2010	6	MCI	18
≥ 2011	16	AD	8
		PD	1
		MS	2

*AD* Alzheimer’s disease, *k* Number of studies, *MCI* Mild cognitive impairment, *MS* Multiple sclerosis, *NDD* Neurodegenerative disease, *PD* Parkinson’s disease

## Appendix 3. Quadas-2 quality assessment and risk of bias

**Table 11:** Signalling questions used to assess risk of bias

**Quadas-2 domain**	**Signalling question(s)**
Patient selection	• Was a consecutive or random sample of patients enrolled? • Was a random sample of controls enrolled or was a longitudinal design used? • Are the patient and control samples comparable with regard to relevant demographic characteristics, or did the study statistically control for significant differences in demographic characteristics? • Did the study avoid inappropriate exclusions?
Index test(s)	• Was the index test appropriate to assess the construct of financial performance? • If a threshold was used, was it pre-specified?
Reference standard	• Was a reference standard used? • Is the reference standard likely to correctly classify the target outcome?
Flow and timing	• Did all participants receive a reference standard? • Did participants receive the same reference standard? • Were all participants included in the analysis?

## Data Availability

The data generated during or analyzed during the current study are available in the DataverseNL repository, https://dataverse.nl/.
